# Supplementary material for: Does cigarette demand respond to price increases in Uganda? Price elasticity estimates using the Uganda National Panel Survey and Deaton’s method
Source: BMJ Open. 2019 Mar 20;9(3):e026150. doi: 10.1136/bmjopen-2018-026150 (PMC6475351; doi:10.1136/bmjopen-2018-026150)
Supplement: Author's manuscript [file bmjopen-2018-026150.draft_revisions.pdf]

BMJ Open

BMJ Open is committed to open peer review. As part of this commitment we make the peer review history of every article we publish publicly available.

When an article is published we post the peer reviewers' comments and the authors' responses online. We also post the versions of the paper that were used during peer review. These are the versions that the peer review comments apply to.

The versions of the paper that follow are the versions that were submitted during the peer review process. They are not the versions of record or the final published versions. They should not be cited or distributed as the published version of this manuscript.

BMJ Open is an open access journal and the full, final, typeset and author-corrected version of record of the manuscript is available on our site with no access controls, subscription charges or pay-per-view fees (http://bmjopen.bmj.com).

If you have any questions on BMJ Open's open peer review process please email info.bmjopen@bmj.com

BMJ Open

Does Cigarette Demand respond to price increases in Uganda? Price Elasticity Estimates using the Uganda National Panel Survey and Deaton's Method

Journal:	*BMJ Open*
Manuscript ID	bmjopen-2018-026150
Article Type:	Research
Date Submitted by the Author:	18-Sep-2018
Complete List of Authors:	Chelwa, Grieve; University of Cape Town, Graduate School of Business<br>Walbeek, Corne; University of Cape Town, Economics
Keywords:	Economics < TROPICAL MEDICINE, Taxation, Price, Public Policy

SCHOLARONE™
Manuscripts

For peer review only

Does Cigarette Demand respond to price increases in Uganda? Price Elasticity Estimates using the Uganda National Panel Survey and Deaton's Method

Authors:
Grieve Chelwa, PhD
Graduate School of Business, University of Cape Town, Cape Town, South Africa.

Corné van Walbeek, PhD
Economics of Tobacco Control Project, School of Economics, University of Cape Town, Cape Town, South Africa.

Corresponding Author:
Grieve Chelwa, PhD
Graduate School of Business
University of Cape Town
Cape Town, South Africa
Email: grievechelwa@gmail.com

World Count: 3,557

For peer review only

ABSTRACT

Objective. To provide the first ever published estimates of the price elasticity of demand for cigarettes in Uganda and thereby contribute to growing the evidence base of the likely impact of excise taxes on cigarette consumption and tax revenues in Sub Saharan Africa.

Method. We use a linear approximation of the Almost Ideal Demand System along with expenditure data from the Uganda National Panel Survey and exploit the fact that prices of cigarettes vary across geographical space in Uganda.

Results. We find that cigarettes are price inelastic in Uganda with elasticity estimates ranging between -0.25 and -0.41. That is, we expect that cigarette demand will decline by between 2.5% and 4.1% every time cigarette prices rise by 10%. These elasticity estimates are in line with international evidence and are robust to outliers in the data.

Conclusion. Our estimates of the price elasticity of demand for cigarettes suggest that the authorities in Uganda can reduce cigarette consumption and simultaneously increase tax revenues by increasing the excise taxes on cigarettes.

Keywords: Economics; Taxation; Public Policy; Price

For peer review only

Strengths and Limitations of this study

- This paper aims at filling the evidence gap for the local impact of excise taxes on cigarette demand in an African setting with specific reference to Uganda.
- There is yet to be a published estimate of the price elasticity of demand for cigarettes in Uganda, a country where excise taxes are low.
- The paper uses the method pioneered by the Nobel Laureate Angus Deaton that exploits the fact that prices of most commodities in developing countries vary across geographical space.
- The biggest limitation of the paper is that it only estimates price elasticities of demand for cigarettes and not all tobacco products in Uganda.
- Another limitation is that the paper only estimates conditional price elasticities (for current cigarette smokers only) and not total price elasticities (conditional plus participation elasticities).

For peer review only

INTRODUCTION

Uganda's smoking prevalence estimates are relatively high by African standards. According to the World Health Organization (WHO), estimates for adult smoking prevalence for males and females were 17% and 3% respectively in 2016 [1]. Comparable estimates for Africa place adult male and female smoking prevalence at 17% and 2% respectively [1]. Among Ugandan youths, smoking prevalence is even higher at 19% for males and 16% for females [2]. There is an expectation that smoking prevalence and tobacco consumption in Uganda will increase in the coming years driven by strong economic growth and declining real prices of tobacco products.

Increasing excise taxes on tobacco products is the single-most effective policy tool for reducing the demand for tobacco products [3, 4]. Unfortunately, excise taxes in Uganda are low and well below the WHO's recommended target. The percentage of the cigarette retail price that is due to the excise tax (known as the excise tax burden) was estimated at 40% in 2017 [2]. WHO recommends an excise tax burden of at least 70% of the retail price [5]. We know from several studies that cigarettes (and all other tobacco products) are generally price inelastic. Available estimates put the price elasticity of demand for cigarettes in the range of -0.20 to -0.60 [3, 4]. The implication of these estimates is that by increasing taxes on cigarettes, countries can decrease consumption and at the same time increase tax revenues.

To the best of our knowledge, there are no published estimates of the price elasticity of demand for cigarettes in Uganda. As a matter of fact, there are very few published estimates of the price

For peer review only

elasticity of demand for cigarettes and tobacco products in general in Africa. The comprehensive survey of the literature on the price elasticity of demand for cigarettes by the International Agency for Research on Cancer was only able to list two sub-Sahara African countries (South Africa and Zimbabwe) as having published estimates of the price elasticity of demand [4].

Even though we expect cigarettes to be price inelastic in most countries, policymakers still demand local evidence before initiating policy changes. There is, therefore, a need to generate local evidence in Africa to support civil society's efforts in encouraging governments to increase the excise tax as part of a comprehensive strategy. This paper contributes to the building of the evidence base on the impact of price/tax measures on cigarette demand in an African setting.

METHOD

This paper uses the method proposed by Deaton [6] and extended in subsequent years [7 - 9] to estimate price elasticities for cigarettes in Uganda. The method uses the fact that prices of most goods in Low- and Middle-Income Countries (LMICs) are similar within narrowly defined clusters like villages but vary across clusters owing to the presence of significant transportation costs as goods move from one cluster to the next. The fact that price variation is largely induced by an external factor (transportation costs) allows one to estimate demand elasticities that are free from concerns of reverse causality or simultaneity bias. Deaton's method has an added layer of attraction for researchers working in Africa because it only uses household expenditure surveys which are available in abundance on the continent. Traditionally, the literature on tobacco demand has estimated price elasticities using aggregate time series data on cigarette

For peer review only

consumption and prices. This partly explains why there are only a handful of countries on the continent with estimates of the price elasticity of demand: many African countries do not maintain time series data of a sufficient time length to allow for a proper estimate of elasticities. On the other hand, many African countries often conduct household expenditure surveys every so often for the purposes of constructing, among other things, Consumer Price Indices (CPIs).

Deaton's method proceeds in a series of steps. First, the researcher extracts unit values (a proxy for prices) from the survey data at the household level. This is done by dividing total expenditure on cigarettes by the quantity demanded of cigarettes or algebraically as

$$v_{ic} = \frac{x_{ic}}{q_{ic}} \quad (1)$$

where v_{ic}, x_{ic} and q_{ic} are respectively the unit value, expenditure and quantity of cigarettes in household i located in cluster c. Deaton uses unit values as opposed to actual prices because household expenditure surveys rarely collect information on the prices faced by individual households. However, unit values are not the same thing as prices. For one thing, unit values hide a great degree of quality heterogeneity whereas the classic treatment of demand concerns itself with homogeneous goods. With quality heterogeneity, households may respond to a price increase by shifting to a lower "quality" brand of cigarettes with a small decline in quantity. Deaton refers to this as "quality shading". With quality shading, the price elasticity of demand will be overestimated. Secondly, unit values are not the same as prices because of measurement

For peer review only

error. Households are unlikely to correctly recall the amount of money spent on cigarettes and/or the quantity consumed. In some cases, the survey enumerator might incorrectly capture this information. In such a case, equation (1) would result in a wrong price even if cigarettes were a homogeneous product (i.e. cigarettes were not susceptible to quality shading). The presence of measurement error will bias the estimate of the price elasticity of demand. Fortunately Deaton has proposed formulae that allow the researcher to correct their final elasticity estimates of quality shading and measurement error (see below).

The second step in Deaton's method involves checking whether the main identifying assumption, that prices (unit values) vary spatially, holds for the unit values obtained above. This is done by using Analysis of Variance (ANOVA) to divide the total variation in unit values into "within cluster variation" and "between cluster variation". A large F statistic for the ANOVA exercise leads to the conclusion that unit values vary across space. In a third step, one estimates within cluster regressions of the form:

$$lnv_{ic} = \lambda + \beta lnx_{ic} + \boldsymbol{\gamma Z_{ic}} + \psi ln\pi_c + e_{ic} \quad (2) \text{ and}$$

$$w_{ic} = \alpha + \varepsilon lnx_{ic} + \boldsymbol{\delta Z_{ic}} + \theta ln\pi_c + (FE_c + u_{ic}) \quad (3)$$

w_{ic} represents the share of cigarette expenditure in total household expenditure for household i in cluster c and lnv_{ic} is the log of the unit value, derived according to equation (1) for household i in cluster c. lnx_{ic} is the log of total household expenditure over the relevant reference period.

$\boldsymbol{Z}_{ic}$ is a vector of household specific characteristics including household size, household gender composition, gender of the household head, proportion of adults in the household and years of schooling of the household head. Other variables in $\boldsymbol{Z}_{ic}$ are the age of the household head and a dummy variable for whether the household head is formally employed or not. FE_c is a cluster fixed effect. u_{ic} and e_{ic} are the standard regression error terms. $ln\pi_c$ are the unobserved prices and consequently, equations (2) and (3) are estimated without them. The coefficients on the price terms can, however, be recovered (see below).

Equation (2), referred to as the "unit value" equation, allows us to check for the presence of quality effects in the unit value data. A positive and statistically significant relationship between household expenditure and unit values, after accounting for household characteristics, would suggest the presence of quality effects. That is, richer households report higher unit values primarily because they are buying cigarettes of a higher quality. Knowing the pattern of the quality effects (i.e. the magnitude of β), allows us to correct our final price elasticity estimates for quality shading. Equation (3), on the other hand, is a standard demand equation where the cigarette share is expressed as a function of household income (proxied by household expenditure), household characteristics and prices. Because of the assumption that prices are fixed within clusters and the fact that we do not have price data, prices are proxied by cluster fixed effects (FE_c). Further, the cluster fixed effects also allow us to control for cluster-level tastes and preferences. Similar tastes and preferences are to be expected for narrowly constructed clusters such as villages. Equations (2) and (3) also contain useful information about measurement error at the household level. The magnitude of the errors are captured by e_{ic} and u_{ic}, the regression error terms. The relationship between the two errors (as captured by, say, the

For peer review only

covariance) is useful in correcting the final price elasticity estimates for measurement error (see more below).

The fourth step involves stripping the household-level demand and unit values of the effects of household expenditure and household characteristics and then averaging across clusters. This step requires the following equations:

$$\widehat{y_c^1} = \frac{1}{n_c}\sum_{i=1}^{n_c}(lnv_{ic} - \hat{\beta} lnx_{ic} - \hat{\boldsymbol{\gamma}}\boldsymbol{Z}_{ic}) \quad (4) \text{ and}$$

$$\widehat{y_c^2} = \frac{1}{n_c}\sum_{i=1}^{n_c}(w_{ic} - \hat{\varepsilon} lnx_{ic} - \hat{\boldsymbol{\delta}}\boldsymbol{Z}_{ic}) \quad (5),$$

where n_c is the number of households in cluster c. $\widehat{y_c^1}$ and $\widehat{y_c^2}$ are the estimates of, respectively, cluster average unit value and cluster average demand after removing the effects of household expenditure and household characteristics (notice that $\widehat{y_c^1}$ and $\widehat{y_c^2}$ do not have the i subscript because they represent cluster averages). Recall that our identifying assumption is that prices vary at the cluster level (i.e. between clusters and not within clusters). Given this identifying assumption, price elasticities of demand can only be obtained by seeing how cluster-level demand responds to changes in cluster-level prices. This leads to the fifth step which involves regressing cluster-level demand, $\widehat{y_c^2}$, on cluster-level unit values, $\widehat{y_c^1}$. The coefficient on $\widehat{y_c^1}$ in such a regression can alternatively be obtained by dividing the covariance between $\widehat{y_c^2}$ and $\widehat{y_c^1}$ by the variance of $\widehat{y_c^1}$. That is $\hat{\phi}$, the estimate of the coefficient on y_c^1, is obtained by

$$\hat{\phi} = \frac{Cov\left(\widehat{y_c^2}, \widehat{y_c^1}\right)}{Var\left(\widehat{y_c^1}\right)} \qquad (6),$$

The sixth and final step in Deaton's method applies quality correction formulas in obtaining the estimate of the price elasticity of demand, $\widehat{\varepsilon_P}$, as follows:

$$\widehat{\varepsilon_P} = \left(\frac{\hat{\theta}}{\bar{w}}\right) - \hat{\psi} \qquad (7),$$

where $\bar{w}$ is the average share of total household expenditure dedicated to cigarettes in the sample. $\hat{\psi}$ and $\hat{\theta}$, the estimates of the coefficients on the unobserved price terms in equations (2) and (3) respectively, are recovered as follows:

$$\hat{\psi} = 1 - \frac{\hat{\beta}(\bar{w} - \hat{\theta})}{\hat{\varepsilon} + \bar{w}} \qquad (8) \text{ and}$$

$$\hat{\theta} = \frac{\hat{\phi}}{1 + (\bar{w} - \hat{\phi})\hat{\zeta}} \qquad (9) \text{ with}$$

$$\hat{\zeta} = \frac{\hat{\beta}}{\hat{\varepsilon} + \bar{w}(1 - \hat{\beta})} \qquad (10).$$

For peer review only

$\hat{\beta}$ is the estimate of the coefficient on total household expenditure in equation (2), the within-cluster unit value equation, and $\hat{\varepsilon}$ is the estimate of the coefficient on total household expenditure in equation (3), the within-cluster demand equation. $\hat{\phi}$ is the estimate of the coefficient of a regression of cluster-level demand on cluster-level unit value (from equation 6).

A small literature has recently emerged that uses Deaton's method in estimating price elasticities of demand for cigarettes in Low- and Middle- Income Countries [10 – 14]. This literature does find price elasticity estimates in line with studies that use the traditional method of time series data.

Data

The data come from the 2005 and 2009 editions of Uganda's National Panel Survey (UNPS), a nationally representative survey. The UNPS is conducted by the Uganda Bureau of Statistics with technical assistance from the World Bank. Households that were surveyed in 2005 were revisited in 2009. Additional panel studies have been conducted since 2009. This paper does not use data from latter rounds because these surveys did not collect sufficiently detailed information on the quantities of cigarettes purchased by households. Specifically, information on whether the unit of purchase was a stick, packet or bundle was missing from the latter rounds of the survey. Deaton's method requires that the units for the quantity variable be the same.

For peer review only

The UNPS collects an exhaustive list of information on the socio-economic characteristics of households. This includes information on household expenditure patterns, household composition patterns, and the occupational and educational status of household members. In the household expenditure module of the survey, which is this paper's primary interest, households are required to recall expenditure on various goods and services. The recall period ranges from 7 days for cigarettes to one year for household expenditures on education, health and clothing to name a few. We convert all expenditures into weekly expenditures.

The expenditure module collects information on the quantity and total amount paid for cigarettes over the last 7 days. The quantities are recorded either as sticks or packets. We convert sticks into packets by dividing them by 20, which is the number of cigarettes in a pack in Uganda. Another unfortunate aspect of the Uganda National Panel Survey is that it does not distinguish between zero expenditures and expenditure information that is simply missing. In other words, a household's information on cigarette expenditure is left out of the survey if (1) the household reported zero expenditure on cigarettes in the previous week or (2) the household was never asked this question or (3) the respondent did not know the answer to the question. Notice that case (1) coincides with a scenario where the household consumes cigarettes but did not do so in the previous week because, say, the price was high (i.e. a corner solution). It also coincides with a scenario where the household does not consume any cigarettes at all. On the other hand, cases (2) and (3) are scenarios where information is simply missing. Because the survey is not explicit about distinguishing zero expenditures from missing expenditures, we choose to only use households that reported positive cigarette expenditures in estimating the price elasticity of demand. In other words, we estimate conditional price elasticities of demand.

For peer review only

In estimating the price elasticity of demand, we run two specifications. The first specification estimates separate price elasticities of demand for the 2005 and 2009 rounds of the Ugandan National Panel Survey. In the second specification, we pool the 2005 and 2009 datasets and estimate a single price elasticity of demand. To allow for comparisons across years, we express all unit values and total expenditures in terms of 2010 prices using Uganda's Consumer Price Index (CPI) [15].

Table 1 reports summary statistics for the relevant variables from the data. We report but do not discuss the summary statistics for the pooled sample because in most cases, the variables for the pooled sample are the averages of the 2005 and 2009 datasets.

For peer review only

Table 1: Summary Statistics from the Uganda National Panel Survey

Variable	2005	2009	Pooled Sample
Percentage of households with positive cigarette expenditure	9%	7%	8%
Average cigarette share in total household expenditure	8.86%	7.67%	8.40%
Real average unit value per pack	1,952.11	1,056.26	1,605.40
Average unit value per pack (USD equivalent)	0.83	0.45	0.68
Average weekly quantity of cigarettes (in packs)	1.45	1.55	1.49
Ave. real h/hold expenditure (last 7 days in Ugandan Shillings)	34,598.08	32,784.07	33,896.02
Ave. household expenditure (last 7 days USD equivalent)	14.72	13.95	14.42
Average household size	5.58	6.33	5.87
Average age of household head in years	41.13	43.73	42.15
Average proportion of male heads	86.81%	90.17%	88.12%
Average proportion of adults	54.58%	46.88%	51.59%
Average proportion of males in household	53.27%	52.00%	52.63%
Average years of schooling of household head	6.55	5.81	6.26
Average proportion of heads with some employment	89.74%	84.43%	87.73%
Total number of clusters	178	121	299
Total number of households	274	173	447
Average number of households per cluster	1.54	1.43	1.49

Notes: Summary statistics for the relevant variables from the 2005 and 2009 Uganda National Panel Survey (UNHS) and the pooled sample. Adults are those household members who are 18 years or older.

8% of the sample of households from 2005 reported positive expenditures on cigarettes in the last 7 days. For 2009, this number was 7%. The share of the weekly budget allocated to cigarette expenditure was, on average, 8.86% in 2005 and 7.67% in 2009. Although Uganda's cigarette expenditure share is relatively high, it falls within the range identified by John [16] particularly for LMICs. The reported average unit value per pack in 2010 Ugandan Shillings was 1,952 (83 US cents) in 2005 and 1,056 (45 US cents) in 2009 [17]. To the extent that unit values are a good proxy of actual prices, then the foregoing would suggest that cigarettes became cheaper, in real terms, by about 46% over the four year period in question. The average weekly household

For peer review only

expenditure expressed in 2010 Uganda shillings was 34,598 (USD15) in 2005 and declined slightly by 5% to 32,784 (USD14) in 2009. The rest of the summary statistics in Table 1 mainly pertain to the ***Z*** vector of control variables used in the regressions (see equations 2 and 3 above).

Patients and public involvement

Patients and the public were not involved in this study.

For peer review only

RESULTS

The main identifying assumption behind Deaton's method is that prices vary across geographical space. The validity of this assumption can be tested using Analysis of Variance (ANOVA) techniques. We report the results of the ANOVA exercise in Table 2. According to the results of the ANOVA procedure, most of the variation in unit values takes place between clusters. The R-squared, which measures the proportion of total variation in unit values between clusters, is at least 70% in all three data specifications. The F statistics associated with a null hypothesis of no spatial variation are all large with the exception of the 2009 sample. Even with the 2009 sample, the R-squared is 72%. In large part, the assumption of spatially varying prices is confirmed by the data. John [10, 11] also finds that cluster effects explain at least 70% of the variation in unit values in India.

Table 2: Testing the Spatial Variation Hypothesis

2005 sample				2009 sample				Pooled sample			
F statistic	p value	R-squared	n	F statistic	p value	R-squared	n	F statistic	p value	R-squared	n
1.29	0.08	0.70	274	1.12	0.33	0.72	173	1.26	0.06	0.72	447

Notes: The F statistic and the p-value are associated with the null hypothesis of no spatial variation in unit values. The hypothesis is rejected in the 2005 and pooled samples but not in the 2009 sample. The R-squared measures the proportion of variation in prices taking place between clusters. n is the total number of households.

Tables 3 and 4 show the results of running the unit value and budget share regressions (equations 2 and 3) on all three samples. The results for the unit value regression are reported in Table 3. The results show that quality effects are present in the data: households with higher household expenditure tend to report higher unit values controlling for other household characteristics and controlling for year effects in the pooled sample. Reported unit values rise by between 1% and 2% for every 10% increase in household expenditure. These so-called expenditure elasticities of quality compare favourably with those estimated for cigarettes by John [10, 11], Eozenou and Fishburn [12], Guindon et al. [13] and Chen and Xing [14].

For peer review only

Table 3: Results of the unit value regression (equation 2)

Variables	(2005) lnv	(2009) lnv	(Pooled) Lnv
lnx	0.234***	0.115**	0.176***
	(0.051)	(0.048)	(0.036)
Size	-0.042	-0.010	-0.019
	(0.124)	(0.119)	(0.088)
Adults	-0.203	0.159	-0.071
	(0.295)	(0.300)	(0.213)
Males	0.261	0.131	0.239
	(0.216)	(0.223)	(0.159)
Education	-0.143*	0.108	-0.023
	(0.080)	(0.074)	(0.055)
Age	-0.015	-0.409**	-0.123
	(0.153)	(0.166)	(0.114)
Gender	0.217	0.218	0.225*
	(0.163)	(0.183)	(0.123)
Work	-0.144	0.101	-0.000
	(0.141)	(0.118)	(0.094)
Year			-0.191***
			(0.061)
Constant	4.957***	6.602***	5.490***
	(0.692)	(0.739)	(0.511)
No. of households	233	147	380
R-squared	0.115	0.126	0.110

Notes: Results of the regression of the log of unit value (lnv) on the log of household expenditure (lnx) and other household characteristics (see equation 2). Household size (Size), education of household head (Education) and age of household head (Age) are in natural logarithms. Adults refers to the proportion of adults in a household and adults are defined as aged 18 years or older. Males is the proportion of males in a household. Gender is a dummy variable which takes on the value of 1 if the household head is male and zero if they are female. Work is a dummy variable which takes on the value of 1 if the household head is employed and zero otherwise. Year takes on the value of 1 if the household was surveyed in 2005 and zero if surveyed in 2009. Standard errors in parentheses. *** p<0.01, ** p<0.05, * p<0.1.

For peer review only

Table 4 reports the results of running the budget share regression (equation 3) on household expenditure alongside other household characteristics, cluster fixed-effects and year effects (for the pooled sample). In Table 4, there is a negative and statistically significant relationship between household expenditure and the share of the household budget allocated to cigarettes. The cigarette budget share tends to fall as household expenditure rises. The cluster fixed effects, whose results are not presented in Table 4 for purposes of space, are all jointly statistically significant at the 1% level for the 2005 and pooled samples and at the 10% level for the 2009 sample. This means that unobserved but fixed factors at the cluster level (prices, tastes, preferences, etc…) matter for cigarette demand. The only other variables that are significant in Table 4 are the gender variable (2009 and pooled sample) and the work dummy (pooled sample).

For peer review only

Table 4: Results of the budget share regression (equation 3)

Variables	(2005) w	(2009) w	(Pooled) w
lnx	-0.056***	-0.065***	-0.056***
	(0.017)	(0.023)	(0.013)
Size	0.002	0.039	0.002
	(0.031)	(0.043)	(0.023)
Adults	0.008	0.092	0.011
	(0.072)	(0.103)	(0.054)
Males	0.013	0.010	0.001
	(0.059)	(0.068)	(0.042)
Education	-0.001	-0.012	-0.007
	(0.020)	(0.025)	(0.015)
Age	0.028	-0.077	0.009
	(0.044)	(0.072)	(0.035)
Gender	-0.038	-0.108*	-0.050*
	(0.037)	(0.056)	(0.029)
Work	0.037	0.058	0.044*
	(0.037)	(0.039)	(0.026)
Year			-0.320***
			(0.107)
Constant	0.533***	0.963***	0.618***
	(0.193)	(0.292)	(0.148)
No. of households	233	147	380
R-squared	0.866	0.909	0.876

Notes: Results of the regression of the cigarette budget share (w) on the log of household expenditure (lnx) and other household characteristics (see equation 3). Household size (Size), education of household head (Education) and age of household head (Age) are in natural logarithms. Adults refers to the proportion of adults in a household and adults are defined as aged 18 years or older. Males is the proportion of males in a household. Gender is a dummy variable which takes on the value of 1 if the household head is male and zero if they are female. Work is a dummy variable which takes on the value of 1 if the household head is employed and zero otherwise. Year takes on the value of 1 if the household was surveyed in 2005 and zero if surveyed in 2009. Standard errors in parentheses. *** p<0.01, ** p<0.05, * p<0.1. Cluster fixed effects are suppressed for space reasons but are jointly statistically significant at the 1% level for the 2005 and pooled samples and at 10% for the 2009 sample.

For peer review only

Table 5 presents our estimates of the price elasticity of demand for cigarettes in Uganda. The estimates are obtained using the information in Tables 3 and 4 alongside the formulae in equations 4 to 10 above. Standard errors (in parenthesis) are obtained by the method of the Bootstrap.

Table 5: Estimates of the price elasticity of demand for cigarettes in Uganda

	(2005)	(2009)	(Pooled sample)
$\widehat{\varepsilon_P}$	-0.326***<br>[0.021]<br>(-0.368 , -0.284)	-0.258***<br>[0.011]<br>(-0.280 , -0.235)	-0.405***<br>[0.048]<br>(-0.499 , -0.311)
No. of households	233	147	380
No. of clusters	184	130	314

Notes: Estimates of the price elasticity of demand for cigarettes in Uganda for the 2005, 2009 and pooled samples. Bootstrapped standard errors are in square brackets. 95% confidence intervals are in parentheses. *** p<0.01, ** p<0.05, * p<0.1.

The estimates in Table 5 show that cigarettes are price inelastic in Uganda. The estimates for the price elasticity of demand for the three data specifications are all statistically significant at the 1% level. For 2005, the price elasticity is estimated at -0.33 while for the 2009 sample the estimate is at -0.26. The pooled sample has a slightly more responsive elasticity at -0.41. Taken together, these results imply that the demand for cigarettes in Uganda is expected to decline by between 3% and 4% for every 10% rise in prices. These results fall within the range of estimates

for studies that use Deaton's method (-0.10 to -0.60) and studies using aggregate time series data (-0.20 to -0.60).

Robustness

The estimates of the price elasticity of demand for cigarettes presented above are likely to be influenced by extreme unit values given that the sample sizes are relatively small. To check the robustness of our estimates, we follow Guindon et al. [13] and exclude unit values that are greater than 2.5 standard deviations and 5 standard deviations from their respective means. (Given the similarity of the results in Tables 5, we only perform the robustness test on the pooled sample).

Table 6 contains the estimates of the price elasticity of demand for cigarettes for the robustness tests. We have also included in Table 6, estimates for the pooled sample taken from Table 5. The second column (column 2) excludes unit values that deviate by more than 5 standard deviations from the mean while the third column (column 3), being more stringent, excludes unit values that deviate by more than 2.5 standard deviations from the mean. The price elasticity estimates in columns 2 and 3 are statistically significant at the 1% level and are similar to the estimate from the pooled sample taken from Table 5.

Table 6: Estimates of price elasticities of demand for cigarettes in Uganda (Robustness check)

	(Pooled sample)	(2)	(3)

For peer review only

$\widehat{\varepsilon_P}$	-0.405***	-0.387***	-0.395***
	[0.048]	[0.068]	[0.082]
	(-0.499, -0.311)	(-0.521 , -0.253)	(-0.555 , -0.235)
No. of households	380	378	375
No. of clusters	314	312	312

Notes: Estimates of the price elasticity of demand for cigarettes in Uganda for the pooled sample and for the pooled sample with extreme unit values excluded. Column 1 (pooled sample) includes all unit values. In column 2, all unit values that are equal to or greater than 5 standard deviations from the mean are excluded. In column 3, all unit values that are equal to or greater than 2.5 standard deviations from the mean are excluded. Bootstrapped standard errors are in square brackets. 95% confidence intervals are in parentheses. *** p<0.01, ** p<0.05, * p<0.1.

For peer review only

DISCUSSION

The aim of this paper was to estimate price elasticities of demand for cigarettes in Uganda. In doing so, we used the Uganda National Panel Survey (UNPS) and exploited the empirical fact that unit values, a measure of prices, varied exogenously across geographical space in Uganda. The last point allowed us to control for possible endogeneity (i.e. reverse causation) in the cigarette demand equation.

We focussed on cigarettes because the first two rounds of the UNPS did not collect detailed enough information on other tobacco products to allow for the estimation of their elasticities using Deaton's method, which was the method we used in the chapter. Further, we were only able to estimate conditional elasticities because the UNPS does not distinguish between households reporting zero expenditure on cigarettes and those whose information is missing.

We found that cigarette demand was price inelastic in Uganda with elasticity estimates ranging between -0.3 to -0.4. This means that the quantity of cigarettes demanded is expected to decline by between 3% and 4%, at the very least, every time prices rise by 10%. Given that these results are conditional elasticities, we expect that the total elasticity is much higher in absolute size since it is the sum of the conditional elasticity (estimated here) and the participation elasticity. Empirically, the total elasticity is about twice the magnitude of the conditional elasticity [4].

Our estimates suggest that Uganda can reduce cigarette consumption by increasing excise taxes on cigarettes. Further, and given that the reduction in demand is expected to be proportionately smaller than the increase in prices, the country can increase excise tax revenues at the same time.

For peer review only

Contributors:

GC did the analysis and wrote-up the paper together with CvW.

Funding:

This work was supported by funding from the Bill and Melinda Gates Foundation.

Competing Interests: None.

Provenance and peer review: Not commissioned; externally peer reviewed

Data sharing statement: All datasets are publicly available.

References

1. World Health Organization. Global Health Observation Data. 2018. Available at: http://www.who.int/gho/tobacco/use/en/ (Accessed June 2018)
2. World Health Organization. Report on the Global Tobacco Epidemic. Geneva: World Health Organization 2017
3. Chaloupka F, Warner KE. The economics of smoking. In: Newhouse J, Cutler D, eds. Handbook of Health Economics. Amsterdam: North Holland 2000: 1539 – 1567
4. International Agency for Research on Cancer. Handbook on the effectiveness of tax and price policies for tobacco control. Geneva: World Health Organization 2011
5. World Health Organization.Technical Manual on Tax Administration. Geneva: World Health Organization 2010
6. Deaton A. Quality, Quantity, and Spatial Variation of Price. American Economic Review 1988; 78: 418 – 430
7. Deaton A. Household Survey Data and Pricing Policies in Developing Countries. World Bank Economic Review 1989; 3: 183 – 210

For peer review only

8. Deaton A. Price elasticities from Survey Data: Extensions and Indonesian Results. Journal of Econometrics 1990; 44: 281 – 304

9. Deaton A. Analysis of Household Surveys. Baltimore: Johns Hopkins Press 1997

10. John RM. Price elasticity estimates for tobacco and other addictive goods in India. Indira Gandhi Institute of Development Research 2005; Working Paper Number WP-2005-003

11. John RM. Price elasticity estimates for tobacco products in India. Health Policy and Planning 2008; 23: 200 – 209

12. Eozenou P, Fishburn B. Price elasticity estimates for cigarette demand in Vietnam. Development and Policies Research Center 2009; Working Paper Number 5

13. Guindon EG, Nandi A, et al. Socioeconomic differences in the impact of smoking tobacco and alcohol prices on smoking in India. National Bureau of Economics Research 2011; Working Paper Number 17580

14. Chen Y, Xing W. Quantity, quality and regional price variation of cigarettes: Demand analysis based on a household survey in China. China Economic Review 2011; 22: 221 – 232

15. We obtain the CPI from the World Development Indicators provided by the World Bank. Available here: http://data.worldbank.org/data-catalog/world-development-indicators

16. John RM. Crowding out effect of tobacco expenditure and its implications on household resource allocation in India. Social Science and Medicine 2008; 66: 1356 – 1367

17. We convert 2010 Uganda shillings into US dollars by using the 2010 end of year exchange rate between the shilling and the dollar available from www.oanda.com.

For peer review only

For peer review only

BMJ Open

Does Cigarette Demand respond to price increases in Uganda? Price Elasticity Estimates using the Uganda National Panel Survey and Deaton's Method

Journal:	*BMJ Open*
Manuscript ID	bmjopen-2018-026150.R1
Article Type:	Research
Date Submitted by the Author:	29-Nov-2018
Complete List of Authors:	Chelwa, Grieve; University of Cape Town, Graduate School of Business<br>Walbeek, Corne; University of Cape Town, Economics
**Primary Subject Heading**:	Smoking and tobacco
Secondary Subject Heading:	Addiction
Keywords:	Economics < TROPICAL MEDICINE, Taxation, Price, Public Policy

SCHOLARONE™ Manuscripts

Does Cigarette Demand respond to price increases in Uganda? Price Elasticity Estimates using the Uganda National Panel Survey and Deaton's Method

Authors:
Grieve Chelwa, PhD
Graduate School of Business, University of Cape Town, Cape Town, South Africa.

Corné van Walbeek, PhD
Economics of Tobacco Control Project, School of Economics, University of Cape Town, Cape Town, South Africa.

Corresponding Author:
Grieve Chelwa, PhD
Graduate School of Business
University of Cape Town
Cape Town, South Africa
Email: grievechelwa@gmail.com

World Count: 3,926

For peer review only

ABSTRACT

Objective. To provide the first ever published estimates of the price elasticity of demand for cigarettes in Uganda and thereby contribute to growing the evidence base of the likely impact of excise taxes on cigarette consumption and tax revenues in Sub Saharan Africa.

Method. We use a linear approximation of the Almost Ideal Demand System along with expenditure data from the Uganda National Panel Survey and exploit the fact that prices of cigarettes vary across geographical space in Uganda.

Results. We find that cigarettes are price inelastic in Uganda with elasticity estimates ranging between -0.26 and -0.33. That is, we expect that cigarette demand will decline by between 2.5% and 3.3% every time cigarette prices rise by 10%. These elasticity estimates are in line with international evidence and are robust to outliers in the data.

Conclusion. Our estimates of the price elasticity of demand for cigarettes suggest that the authorities in Uganda can reduce cigarette consumption and simultaneously increase tax revenues by increasing the excise taxes on cigarettes.

Keywords: Economics; Taxation; Public Policy; Price

For peer review only

Strengths and Limitations of this study

- This paper aims at filling the evidence gap for the local impact of excise taxes on cigarette demand in an African setting with specific reference to Uganda.
- There is yet to be a published estimate of the price elasticity of demand for cigarettes in Uganda, a country where excise taxes are low.
- The paper uses the method pioneered by Angus Deaton that exploits the fact that prices of most commodities in developing countries vary across geographical space.
- A limitation is that the paper only estimates conditional price elasticities (for current cigarette smokers only) and not total price elasticities (conditional plus participation elasticities).

For peer review only

INTRODUCTION

Uganda's smoking prevalence estimates are relatively high by African standards. According to the World Health Organization (WHO), estimates for adult smoking prevalence for males and females were 17% and 3% respectively in 2016 [1]. Comparable estimates for Africa place adult male and female smoking prevalence at 17% and 2% respectively [1]. Among Ugandan youths, smoking prevalence is even higher at 19% for males and 16% for females [2]. Prevalence is relatively high in Uganda in comparison to other African countries because Uganda has, in the recent past, experienced relatively impressive economic growth with a modest increase in the real price of tobacco products. For example, Ho et al. [3] estimate that Gross National Income Per Capita increased by 107% between 1999 and 2013. Over the same period, real cigarette prices only increased by 42% [3]. Therefore, smoking prevalence and tobacco consumption in Uganda will increase in the coming years if these trends continue.

Increasing excise taxes on tobacco products is the single-most effective policy tool for reducing the demand for tobacco products [4, 5]. Unfortunately, excise taxes in Uganda are low and well below the WHO's recommended target. The percentage of the cigarette retail price that is due to the excise tax (known as the excise tax burden) was estimated at 40% in 2017 [2]. WHO recommends an excise tax burden of at least 70% of the retail price [6]. We know from several studies that cigarettes (and all other tobacco products) are generally price inelastic. Available estimates put the price elasticity of demand for cigarettes in the range of -0.20 to -0.60 [4, 5]. The implication of these estimates is that by increasing taxes on cigarettes, countries can decrease consumption and at the same time increase tax revenues.

For peer review only

To the best of our knowledge, there are no published estimates of the price elasticity of demand for cigarettes that focus specifically on Uganda. As a matter of fact, there are very few published estimates of the price elasticity of demand for cigarettes and tobacco products in Africa. The comprehensive survey of the literature on the price elasticity of demand for cigarettes by the International Agency for Research on Cancer was only able to list two sub-Sahara African countries (South Africa and Zimbabwe) as having published estimates of the price elasticity of demand [5]. These studies found price elasticity estimates ranging between -0.16 and -1.52. Using panel data techniques, Ho et al. [3] have recently provided estimates of the price elasticity of demand for cigarettes for the African continent using a sample of 36 countries. Their full-sample estimate of -0.50 is right within the range of international estimates [4, 5]. Much like the international literature, Ho et al. [3] show that there is heterogeneity in elasticity estimates by income category. Low-income African countries have, as a group, the highest elasticity estimates with high-income countries having the lowest estimates.

Even though we expect cigarettes to be price inelastic in most countries, policymakers still demand local evidence before initiating policy changes. There is, therefore, a need to generate local evidence in Africa to support civil society's efforts in encouraging governments to increase the excise tax as part of a comprehensive strategy. Often generating local evidence is constrained by the unavailability of appropriate data. The method used in this paper utilizes expenditure data, widely available on the continent, to estimate elasticities. The fact that expenditure data are widely available on the African continent means that Deaton's method holds much promise for generating local evidence on the continent. A small literature has recently emerged that uses Deaton's method

in estimating price elasticities of demand for cigarettes in Low- and Middle- Income Countries [7 – 11]. This literature's price elasticity estimates (-0.10 to -0.60) are in line with studies that use more traditional time series methods (-0.20 to -0.60). This method is yet to be applied to an African country and Uganda provides us with a unique opportunity to demonstrate the efficacy of the method.

METHOD

This paper uses the method proposed by Deaton [12] and extended in subsequent years [13 - 15] to estimate price elastcities for cigarettes in Uganda. The method uses the fact that prices of most goods in Low- and Middle-Income Countries (LMICs) are similar within narrowly defined clusters like villages but vary across clusters owing to the presence of significant transportation costs as goods move from one cluster to the next. The fact that price variation is largely induced by an external factor (transportation costs) allows one to estimate demand elasticities that are free from concerns of reverse causality or simultaneity bias. Deaton's method has an added layer of attraction for researchers working in Africa because it only uses household expenditure surveys which are available in abundance on the continent. Traditionally, the literature on tobacco demand has estimated price elasticities using aggregate time series data on cigarette consumption and prices. This partly explains why there are only a handful of countries on the continent with estimates of the price elasticity of demand: many African countries do not maintain time series data of a sufficient time length to allow for a proper estimate of elasticities. On the other hand, many African countries often conduct household expenditure surveys every so often for the purposes of constructing, among other things, Consumer Price Indices (CPIs).

For peer review only

Deaton's method proceeds in a series of steps. First, the researcher extracts unit values (a proxy for prices) from the survey data at the household level. This is done by dividing total expenditure on cigarettes by the quantity demanded of cigarettes or algebraically as

$$v_{ic} \equiv \frac{x_{ic}}{q_{ic}} \qquad (1)$$

where v_{ic}, x_{ic} and q_{ic} are respectively the unit value, expenditure and quantity of cigarettes in household i located in cluster c. Deaton uses unit values as opposed to actual prices because household expenditure surveys rarely collect information on the prices faced by individual households. However, unit values are not the same thing as prices. For one thing, unit values hide a great degree of quality heterogeneity whereas the classic treatment of demand concerns itself with homogeneous goods. With quality heterogeneity, households may respond to a price increase by shifting to a lower "quality" brand of cigarettes with a small decline in quantity. Deaton refers to this as "quality shading". With quality shading, the price elasticity of demand will be overestimated. Secondly, unit values are not the same as prices because of measurement error. Households are unlikely to correctly recall the amount of money spent on cigarettes and/or the quantity consumed. In some cases, the survey enumerator might incorrectly capture this information. In such a case, (1) would result in a wrong price even if cigarettes were a homogeneous product (i.e. cigarettes were not susceptible to quality shading). The presence of measurement error will bias the estimate of the price elasticity of demand. Fortunately Deaton has

For peer review only

proposed formulae that allow the researcher to correct their final elasticity estimates of quality shading and measurement error (see below).

The second step in Deaton's method involves checking whether the main identifying assumption, that prices (unit values) vary spatially, holds for the unit values obtained above. This is done by using Analysis of Variance (ANOVA) to divide the total variation in unit values into "within cluster variation" and "between cluster variation". A large F statistic for the ANOVA exercise leads to the conclusion that unit values vary across space. In a third step, one estimates within cluster regressions of the form:

$$lnv_{ic} = \lambda + \beta lnx_{ic} + \boldsymbol{\gamma Z_{ic}} + \psi ln\pi_c + e_{ic} \qquad (2) \text{ and}$$

$$w_{ic} = \alpha + \varepsilon lnx_{ic} + \boldsymbol{\delta Z_{ic}} + \theta ln\pi_c + (FE_c + u_{ic}) \qquad (3)$$

w_{ic} represents the share of cigarette expenditure in total household expenditure for household i in cluster c and lnv_{ic} is the log of the unit value, derived according to identity (1) for household i in cluster c. lnx_{ic} is the log of total household expenditure over the relevant reference period. $\boldsymbol{Z_{ic}}$ is a vector of household specific characteristics including household size, household gender composition, gender of the household head, proportion of adults in the household and years of schooling of the household head. Other variables in $\boldsymbol{Z_{ic}}$ are the age of the household head and a dummy variable for whether the household head is formally employed or not. FE_c is a cluster fixed effect. u_{ic} and e_{ic} are the standard regression error terms. $ln\pi_c$ are the unobserved prices and

For peer review only

consequently, equations (2) and (3) are estimated without them. The coefficients on the price terms can, however, be recovered (see below).

Equation (2), referred to as the "unit value" equation, allows us to check for the presence of quality effects in the unit value data. A positive and statistically significant relationship between household expenditure and unit values, after accounting for household characteristics, would suggest the presence of quality effects. That is, richer households report higher unit values primarily because they are buying cigarettes of a higher quality. Knowing the pattern of the quality effects (i.e. the magnitude of β), allows us to correct our final price elasticity estimates for quality shading. It is also important to note that some portion of the variation in unit values comes from these choices in quality that households are making. This variation is also exploited below in estimating our elasticities. Equation (3), on the other hand, is a standard demand equation where the cigarette share is expressed as a function of household income (proxied by household expenditure), household characteristics and prices. The cluster fixed effects allow us to control for cluster-level tastes and preferences. Similar tastes and preferences are to be expected for narrowly constructed clusters such as villages. Equations (2) and (3) also contain useful information about measurement error at the household level. The magnitude of the errors are captured by e_{ic} and u_{ic}, the regression error terms. The relationship between the two errors (as captured by, say, the covariance) is useful in correcting the final price elasticity estimates for measurement error (see more below).

For peer review only

The fourth step involves stripping the household-level demand and unit values of the effects of household expenditure and household characteristics and then averaging across clusters. This step requires the following equations:

$$\widehat{y_c^1} = \frac{1}{n_c}\sum_{i=1}^{n_c}(lnv_{ic} - \hat{\beta}lnx_{ic} - \hat{\boldsymbol{\gamma}}\boldsymbol{Z}_{ic}) \qquad (4) \text{ and}$$

$$\widehat{y_c^2} = \frac{1}{n_c}\sum_{i=1}^{n_c}(w_{ic} - \hat{\varepsilon}lnx_{ic} - \hat{\boldsymbol{\delta}}\boldsymbol{Z}_{ic}) \qquad (5),$$

where n_c is the number of households in cluster c. $\widehat{y_c^1}$ and $\widehat{y_c^2}$ are the estimates of, respectively, cluster average unit value and cluster average demand after removing the effects of household expenditure and household characteristics (notice that $\widehat{y_c^1}$ and $\widehat{y_c^2}$ do not have the i subscript because they represent cluster averages). Recall that our identifying assumption is that prices vary at the cluster level (i.e. between clusters and not within clusters). Given this identifying assumption, price elasticities of demand can only be obtained by seeing how cluster-level demand responds to changes in cluster-level prices. This leads to the fifth step which involves regressing cluster-level demand, $\widehat{y_c^2}$, on cluster-level unit values, $\widehat{y_c^1}$. The coefficient on $\widehat{y_c^1}$ in such a regression can alternatively be obtained by dividing the covariance between $\widehat{y_c^2}$ and $\widehat{y_c^1}$ by the variance of $\widehat{y_c^1}$. That is $\hat{\phi}$, the estimate of the coefficient on y_c^1, is obtained by

$$\hat{\phi} = \frac{Cov\left(\widehat{y_c^2}, \widehat{y_c^1}\right) - \frac{\widehat{\sigma^{12}}}{n_c}}{Var\left(\widehat{y_c^1}\right) - \frac{\widehat{\sigma^{11}}}{n_c^+}} \qquad (6),$$

For peer review only

where n_c^+ is the number of households in a village reporting positive expenditures on tobacco and n_c is the number of households in a village; $\widehat{\sigma^{12}}$ is the estimate of the covariance of the errors in equations (2) and (3); $\widehat{\sigma^{11}}$ is the variance of the errors in equation (2). These are included in equation (6) to adjust our estimates for measurement error.

The sixth and final step in Deaton's method applies quality correction formulas in obtaining the estimate of the price elasticity of demand, $\widehat{\varepsilon_P}$, as follows:

$$\widehat{\varepsilon_P} = \left(\frac{\hat{\theta}}{\overline{\overline{w}}}\right) - \hat{\psi} \qquad (7),$$

where $\overline{w}$ is the average share of total household expenditure dedicated to cigarettes in the sample. $\hat{\psi}$ and $\hat{\theta}$, the estimates of the coefficients on the unobserved price terms in equations (2) and (3) respectively, are recovered as follows:

$$\hat{\psi} = 1 - \frac{\hat{\beta}(\overline{w} - \hat{\theta})}{\hat{\varepsilon} + \overline{w}} \qquad (8) \text{ and}$$

$$\hat{\theta} = \frac{\hat{\phi}}{1 + (\overline{w} - \hat{\phi})\hat{\zeta}} \qquad (9) \text{ with}$$

For peer review only

$$\hat{\zeta} = \frac{\hat{\beta}}{\hat{\varepsilon} + \bar{w}(1 - \hat{\beta})} \qquad (10).$$

$\hat{\beta}$ is the estimate of the coefficient on total household expenditure in equation (2), the within-cluster unit value equation, and $\hat{\varepsilon}$ is the estimate of the coefficient on total household expenditure in equation (3), the within-cluster demand equation. $\hat{\phi}$ is the estimate of the coefficient of a regression of cluster-level demand on cluster-level unit value (from equation 6).

Deaton also proposes the following formula for obtaining the estimate of the expenditure elasticity of demand, $\widehat{\varepsilon_I}$:

$$\widehat{\varepsilon_I} = 1 + \left(\frac{\hat{\varepsilon}}{\bar{w}}\right) - \hat{\beta} \qquad (3.11).$$

Data

The data come from the 2005 and 2009 editions of Uganda's National Panel Survey (UNPS), a nationally representative survey. The UNPS is conducted by the Uganda Bureau of Statistics with technical assistance from the World Bank. Households that were surveyed in 2005 were revisited in 2009. Additional panel studies have been conducted since 2009. This paper does not use data from latter rounds because these surveys did not collect sufficiently detailed information on the quantities of cigarettes purchased by households. Specifically, information on whether the unit of purchase was a stick, packet or bundle was missing from the latter rounds of the survey. Deaton's

For peer review only

method requires that the units for the quantity variable be the same. Further, we do not exploit the panel characteristics of the paper but instead treat 2005 and 2009 as separate cross-sections.

The UNPS collects an exhaustive list of information on the socio-economic characteristics of households. This includes information on household expenditure patterns, household composition patterns, and the occupational and educational status of household members. In the household expenditure module of the survey, which is this paper's primary interest, households are required to recall expenditure on various goods and services. The recall period ranges from 7 days for cigarettes to one year for household expenditures on education, health and clothing to name a few. We convert all expenditures into weekly expenditures.

The expenditure module collects information on the quantity and total amount paid for cigarettes over the last 7 days. The quantities are recorded either as sticks or packets. We convert sticks into packets by dividing them by 20, which is the number of cigarettes in a pack in Uganda. Another unfortunate aspect of the Uganda National Panel Survey is that it does not distinguish between zero expenditures and expenditure information that is simply missing. In other words, a household's information on cigarette expenditure is left out of the survey if (1) the household reported zero expenditure on cigarettes in the previous week or (2) the household was never asked this question or (3) the respondent did not know the answer to the question. Notice that case (1) coincides with a scenario where the household consumes cigarettes but did not do so in the previous week because, say, the price was high (i.e. a corner solution). It also coincides with a scenario where the household does not consume any cigarettes at all. On the other hand, cases (2) and (3) are scenarios where information is simply missing. Because the survey is not explicit about

For peer review only

distinguishing zero expenditures from missing expenditures, we choose to only use households that reported positive cigarette expenditures in estimating the price elasticity of demand. In other words, we estimate conditional price elasticities of demand. To allow for comparisons across years, we express all unit values and total expenditures in terms of 2010 prices using Uganda's Consumer Price Index (CPI) [16].

Table 1 reports summary statistics for the relevant variables from the data.

Table 1: Summary Statistics from the Uganda National Panel Survey

Variable	2005	2009
Percentage of households with positive cigarette expenditure	9%	7%
Average cigarette share in total household expenditure	8.86%	7.67%
Real average unit value per pack	1,952.11	1,056.26
Average unit value per pack (USD equivalent)	0.83	0.45
Average weekly quantity of cigarettes (in packs)	1.45	1.55
Ave. real h/hold expenditure (last 7 days in Ugandan Shillings)	34,598.08	32,784.07
Ave. household expenditure (last 7 days USD equivalent)	14.72	13.95
Average household size	5.58	6.33
Average age of household head in years	41.13	43.73
Average proportion of male heads	86.81%	90.17%
Average proportion of adults	54.58%	46.88%
Average proportion of males in household	53.27%	52.00%
Average years of schooling of household head	6.55	5.81
Average proportion of heads with some employment	89.74%	84.43%
Total number of clusters	322	318
Total number of effective clusters	178	121
Total number of households	274	173
Average number of households per cluster	1.54	1.43

Notes: Summary statistics for the relevant variables from the 2005 and 2009 Uganda National Panel Survey (UNHS). Adults are those household members who are 18 years or older.

For peer review only

8% of the sample of households from 2005 reported positive expenditures on cigarettes in the last 7 days. For 2009, this number was 7%. The share of the weekly budget allocated to cigarette expenditure was, on average, 8.86% in 2005 and 7.67% in 2009. Although Uganda's cigarette expenditure share is relatively high, it falls within the range identified by John [17] particularly for LMICs. The reported average unit value per pack in 2010 Ugandan Shillings was 1,952 (83 US cents) in 2005 and 1,056 (45 US cents) in 2009 [18]. To the extent that unit values are a good proxy of actual prices, then the foregoing would suggest that cigarettes became cheaper, in real terms, by about 46% over the four year period in question. The average weekly household expenditure expressed in 2010 Uganda shillings was 34,598 (USD15) in 2005 and declined slightly by 5% to 32,784 (USD14) in 2009. The rest of the summary statistics in Table 1 mainly pertain to the ***Z*** vector of control variables used in the regressions (see equations 2 and 3 above).

Patients and public involvement

Patients and the public were not involved in this study.

RESULTS

The main identifying assumption behind Deaton's method is that prices vary across geographical space. The validity of this assumption can be tested using Analysis of Variance (ANOVA) techniques. We report the results of the ANOVA exercise in Table 2. According to the results of the ANOVA procedure, most of the variation in unit values takes place between clusters. The R-squared, which measures the proportion of total variation in unit values between clusters, is at least 70% in all three data specifications. The F statistics associated with a null hypothesis of no spatial variation are all large with the exception of the 2009 sample. Even with the 2009 sample, the R-

For peer review only

squared is 72%. In large part, the assumption of spatially varying prices is confirmed by the data. John [7, 8] also finds that cluster effects explain at least 70% of the variation in unit values in India.

Table 2: Testing the Spatial Variation Hypothesis

2005 sample				2009 sample			
F statistic	p value	R-squared	n	F statistic	p value	R-squared	n
1.29	0.08	0.70	274	1.12	0.33	0.72	173

Notes: The F statistic and the p-value are associated with the null hypothesis of no spatial variation in unit values. The hypothesis is rejected in the 2005 sample but not in the 2009 sample. The R-squared measures the proportion of variation in prices taking place between clusters. n is the total number of households.

Tables 3 and 4 show the results of running the unit value and budget share regressions (equations 2 and 3) on the two samples. The results for the unit value regression are reported in Table 3. The results show that quality effects are present in the data: households with higher household expenditure tend to report higher unit values controlling for other household characteristics and controlling for year effects in the pooled sample. Reported unit values rise by between 1% and 2% for every 10% increase in household expenditure. These so-called expenditure elasticities of quality compare favourably with those estimated for cigarettes by John [7, 8], Eozenou and Fishburn [9], Guindon et al. [10] and Chen and Xing [11].

For peer review only

Table 3: Results of the unit value regression (equation 2)

Variables	(2005) lnv	(2009) Lnv
lnx	0.234***	0.115**
	(0.051)	(0.048)
Size	-0.042	-0.010
	(0.124)	(0.119)
Adults	-0.203	0.159
	(0.295)	(0.300)
Males	0.261	0.131
	(0.216)	(0.223)
Education	-0.143*	0.108
	(0.080)	(0.074)
Age	-0.015	-0.409**
	(0.153)	(0.166)
Gender	0.217	0.218
	(0.163)	(0.183)
Work	-0.144	0.101
	(0.141)	(0.118)
Year		
Constant	4.957***	6.602***
	(0.692)	(0.739)
No. of households	233	147
R-squared	0.115	0.126

Notes: Results of the regression of the log of unit value (lnv) on the log of household expenditure (lnx) and other household characteristics (see equation 2). Household size (Size), education of household head (Education) and age of household head (Age) are in natural logarithms. Adults refers to the proportion of adults in a household and adults are defined as aged 18 years or older. Males is the proportion of males in a household. Gender is a dummy variable which takes on the value of 1 if the household head is male and zero if they are female. Work is a dummy variable which takes on the value of 1 if the household head is employed and zero otherwise.. Standard errors in parentheses. *** p<0.01, ** p<0.05, * p<0.1.

For peer review only

Table 4 reports the results of running the budget share regression (equation 3) on household expenditure alongside other household characteristics and cluster fixed-effects. In Table 4, there is a negative and statistically significant relationship between household expenditure and the share of the household budget allocated to cigarettes. The cigarette budget share tends to fall as household expenditure rises. The cluster fixed effects, whose results are not presented in Table 4 for purposes of space, are all jointly statistically significant at the 1% level for the 2005 and at the 10% level for the 2009 sample. This means that unobserved but fixed factors at the cluster level (tastes, preferences, etc…) matter for cigarette demand.

For peer review only

Table 4: Results of the budget share regression (equation 3)

Variables	(2005) w	(2009) w
lnx	-0.056***	-0.065***
	(0.017)	(0.023)
Size	0.002	0.039
	(0.031)	(0.043)
Adults	0.008	0.092
	(0.072)	(0.103)
Males	0.013	0.010
	(0.059)	(0.068)
Education	-0.001	-0.012
	(0.020)	(0.025)
Age	0.028	-0.077
	(0.044)	(0.072)
Gender	-0.038	-0.108*
	(0.037)	(0.056)
Work	0.037	0.058
	(0.037)	(0.039)
Year		
Constant	0.533***	0.963***
	(0.193)	(0.292)
No. of households	233	147
R-squared	0.866	0.909

Notes: Results of the regression of the cigarette budget share (w) on the log of household expenditure (lnx) and other household characteristics (see equation 3). Household size (Size), education of household head (Education) and age of household head (Age) are in natural logarithms. Adults refers to the proportion of adults in a household and adults are defined as aged 18 years or older. Males is the proportion of males in a household. Gender is a dummy variable which takes on the value of 1 if the household head is male and zero if they are female. Work is a dummy variable which takes on the value of 1 if the household head is employed and zero otherwise. Standard errors in parentheses. *** p<0.01, ** p<0.05, * p<0.1. Cluster fixed effects are suppressed for space reasons but are jointly statistically significant at the 1% level for the 2005 sample and at 10% for the 2009 sample.

For peer review only

Table 5 presents our estimates of the price elasticity of demand for cigarettes in Uganda. The estimates are obtained using the information in Tables 3 and 4 alongside the formulae in equations 4 to 10 above. Standard errors (in parenthesis) are obtained by the method of the Bootstrap.

Table 5: Estimates of the price elasticity of demand for cigarettes in Uganda

	**(2005)**	**(2009)**
$\hat{\varepsilon}_P$	-0.326***<br>[0.021]<br>(-0.368 , -0.284)	-0.258***<br>[0.011]<br>(-0.280 , -0.235)
No. of households	233	147
No. of clusters	184	130

Notes: Estimates of the price elasticity of demand for cigarettes in Uganda for the 2005, 2009 and pooled samples. Bootstrapped standard errors are in square brackets. 95% confidence intervals are in parentheses. *** p<0.01, ** p<0.05, * p<0.1.

The estimates in Table 5 show that cigarettes are price inelastic in Uganda. The estimates for the price elasticity of demand for the two samples are all statistically significant at the 1% level. For 2005, the price elasticity is estimated at -0.33 while for the 2009 sample the estimate is at -0.26. Taken together, these results imply that the demand for cigarettes in Uganda is expected to decline by about 3% for every 10% rise in prices. These results fall within the range of estimates for studies that use Deaton's method (-0.10 to -0.60) and studies using aggregate time series data (-0.20 to -0.60).

Table 6 presents estimates of the expenditure elasticity of demand using equation (11) and coefficient estimates from Tables 3 and 4. The estimates in Table 6 show that cigarettes are normal

For peer review only

goods in Uganda. That is, cigarette demand does not, at the very least, decline with an increase in household income (expenditure).

Table 6: Estimates of expenditure elasticities of demand for cigarettes in Uganda

	**(2005)**	**(2009)**
$\hat{\varepsilon}_I$	0.132<br>[0.338]<br>(-0.531 , 0.796)	0.043<br>[0.539]<br>(-1.014 , 1.100)
No. of households	233	147

Notes: Estimates of the expenditure elasticity of demand for cigarettes in Uganda for the 2005 and 2009 samples. Bootstrapped standard errors are in square brackets. 95% confidence intervals are in parentheses. Since the expenditure elasticity of demand is estimated at the household level (see equation 11), we only report the number of households.

Robustness

The estimates of the price elasticity of demand for cigarettes presented above are likely to be influenced by extreme unit values given that the sample sizes are relatively small. To check the robustness of our estimates, we follow Guindon et al. [10] and exclude unit values that are greater than 2.5 standard deviations and 5 standard deviations from their respective means. (Given the similarity of the results in Tables 5 and the better performance of the 2005 sample in the ANOVA exercise, we only perform the robustness test on the 2005 sample).

Table 7 contains the estimates of the price elasticity of demand for cigarettes for the robustness tests. We have also included in Table 7, estimates for the 2005 sample taken from Table 5. The second column (column 2) excludes unit values that deviate by more than 5 standard deviations

For peer review only

from the mean while the third column (column 3), being more stringent, excludes unit values that deviate by more than 2.5 standard deviations from the mean. The price elasticity estimates in columns 2 and 3 are statistically significant at the 1% level and 5% level respectively and are similar to the estimate from the 2005 sample taken from Table 5.

Table 7: Estimates of price elasticities of demand for cigarettes in Uganda (Robustness check)

	(2005 Full sample)	(2)	(3)
$\hat{\varepsilon}_P$	-0.326***	-0.351***	-0.271**
	[0.021]	[0.054]	[0.120]
	(-0.368, -0.284)	(-0.458 , -0.246)	(-0.526 , -0.017)
No. of households	233	231	227
No. of clusters	184	176	175

Notes: Estimates of the price elasticity of demand for cigarettes in Uganda for the 2005 sample and for the 2005 sample with extreme unit values excluded. Column 1 (2005 sample) includes all unit values. In column 2, all unit values that are equal to or greater than 5 standard deviations from the mean are excluded. In column 3, all unit values that are equal to or greater than 2.5 standard deviations from the mean are excluded. Bootstrapped standard errors are in square brackets. 95% confidence intervals are in parentheses. *** p<0.01, ** p<0.05, * p<0.1.

DISCUSSION

This paper had two objectives in mind. First, to provide the first ever estimates of the price elasticity of demand for cigarettes in Uganda, a country where prevalence and consumption are expected to increase in the future. The experience in other African countries, particularly South Africa [19], shows that consistently increasing excise taxes can play a significant role in reducing consumption. The evidence generated in this paper will be useful for policy makers and civil society actors involved in tobacco control efforts in Uganda. The second aim for the paper was to demonstrate the efficacy of using Deaton's method in an African setting where the unavailability

For peer review only

of appropriate data has in the past prevented the estimation of price elasticities. Deaton's method relies on expenditure data which is widely available across the African continent. This, alongside with the fact that our estimates for Uganda are in line with international evidence [3 – 5] and in line with studies using Deaton's method [7 -11], will hopefully encourage other researchers working on the continent to consider using this method for generating local evidence.

Our study focussed on cigarettes because the first two rounds of the UNPS did not collect detailed enough information on other tobacco products to allow for the estimation of their elasticities using Deaton's method. Further, we were only able to estimate conditional elasticities because the UNPS does not distinguish between households reporting zero expenditure on cigarettes and those whose information is missing. Therefore, the total price response of cigarettes in Uganda is likely to be twice our estimated conditional elasticity of -0.30 given the empirical finding that the total elasticity is often double the magnitude of the conditional elasticity [5]. This is similar to the finding in Ho et al. [3] where the [total] price elasticity of low-income African countries, in which Uganda is categorized, is estimated at -0.56.

Given that our study only focussed on cigarettes in Uganda, future research should aim at estimating price elasticities for other tobacco products and cross-price elasticities between cigarettes and other types of tobacco products . Cross price elasticities are important to know given the fact that levying taxes on cigarettes might not only affect cigarette demand, but could also increase the demand for other types of tobacco products. This, however, requires that the statistical

For peer review only

authorities in Uganda consider collecting expenditure information on other types of tobacco products.

Contributors:

GC did the analysis and wrote-up the paper together with CvW.

Funding:

This work was supported by funding from the Bill and Melinda Gates Foundation.

Competing Interests: None.

Provenance and peer review: Not commissioned; externally peer reviewed

Data sharing statement: All datasets are publicly available.

For peer review only

References

1. World Health Organization. Global Health Observation Data. 2018. Available at: http://www.who.int/gho/tobacco/use/en/ (Accessed June 2018)
2. World Health Organization. Report on the Global Tobacco Epidemic. Geneva: World Health Organization 2017
3. Ho L-M, Schafferer C, et al. The effect of cigarette price increases on cigarette consumption, tax revenue, and smoking-related death in Africa from 1999 to 2013. International Journal of Public Health 2017; 8: 899 – 909
4. Chaloupka F, Warner KE. The economics of smoking. In: Newhouse J, Cutler D, eds. Handbook of Health Economics. Amsterdam: North Holland 2000: 1539 – 1567
5. International Agency for Research on Cancer. Handbook on the effectiveness of tax and price policies for tobacco control. Geneva: World Health Organization 2011
6. World Health Organization.Technical Manual on Tax Administration. Geneva: World Health Organization 2010
7. John RM. Price elasticity estimates for tobacco and other addictive goods in India. Indira Gandhi Institute of Development Research 2005; Working Paper Number WP-2005-003
8. John RM. Price elasticity estimates for tobacco products in India. Health Policy and Planning 2008; 23: 200 – 209
9. Eozenou P, Fishburn B. Price elasticity estimates for cigarette demand in Vietnam. Development and Policies Research Center 2009; Working Paper Number 5
10. Guindon EG, Nandi A, et al. Socioeconomic differences in the impact of smoking tobacco and alcohol prices on smoking in India. National Bureau of Economics Research 2011; Working Paper Number 17580

For peer review only

11. Chen Y, Xing W. Quantity, quality and regional price variation of cigarettes: Demand analysis based on a household survey in China. China Economic Review 2011; 22: 221 – 232

12. Deaton A. Analysis of Household Surveys. Baltimore: Johns Hopkins Press 1997

13. Deaton A. Quality, Quantity, and Spatial Variation of Price. American Economic Review 1988; 78: 418 – 430

14. Deaton A. Household Survey Data and Pricing Policies in Developing Countries. World Bank Economic Review 1989; 3: 183 – 210

15. Deaton A. Price elasticities from Survey Data: Extensions and Indonesian Results. Journal of Econometrics 1990; 44: 281 – 304

16. We obtain the CPI from the World Development Indicators provided by the World Bank. Available here: http://data.worldbank.org/data-catalog/world-development-indicators

17. John RM. Crowding out effect of tobacco expenditure and its implications on household resource allocation in India. Social Science and Medicine 2008; 66: 1356 – 1367

18. We convert 2010 Uganda shillings into US dollars by using the 2010 end of year exchange rate between the shilling and the dollar available from www.oanda.com.

19. Chelwa G, Van Walbeek C, Blecher E. Evaluating South Africa's tobacco control policy using a synthetic control method. Tobacco Control 2017; 26: 509 – 517

For peer review only

BMJ Open

Does Cigarette Demand respond to price increases in Uganda? Price Elasticity Estimates using the Uganda National Panel Survey and Deaton's Method

Journal:	*BMJ Open*
Manuscript ID	bmjopen-2018-026150.R2
Article Type:	Research
Date Submitted by the Author:	22-Jan-2019
Complete List of Authors:	Chelwa, Grieve; University of Cape Town, Graduate School of Business<br>Walbeek, Corne; University of Cape Town, Economics
**Primary Subject Heading**:	Smoking and tobacco
Secondary Subject Heading:	Addiction
Keywords:	Economics < TROPICAL MEDICINE, Taxation, Price, Public Policy

SCHOLARONE™
Manuscripts

Does Cigarette Demand respond to price increases in Uganda? Price Elasticity Estimates using the Uganda National Panel Survey and Deaton's Method

Authors:
Grieve Chelwa, PhD
Graduate School of Business, University of Cape Town, Cape Town, South Africa.

Corné van Walbeek, PhD
Economics of Tobacco Control Project, School of Economics, University of Cape Town, Cape Town, South Africa.

Corresponding Author:
Grieve Chelwa, PhD
Graduate School of Business
University of Cape Town
Cape Town, South Africa
Email: grievechelwa@gmail.com

World Count: 3,926

For peer review only

ABSTRACT

Objective. To provide the first ever published estimates of the price elasticity of demand for cigarettes in Uganda and thereby contribute to growing the evidence base of the likely impact of excise taxes on cigarette consumption and tax revenues in Sub Saharan Africa.

Method. We use a linear approximation of the Almost Ideal Demand System along with expenditure data from the Uganda National Panel Survey and exploit the fact that prices of cigarettes vary across geographical space in Uganda.

Results. We find that cigarettes are price inelastic in Uganda with elasticity estimates ranging between -0.26 and -0.33. That is, we expect that cigarette demand will decline by between 2.5% and 3.3% every time cigarette prices rise by 10%. These elasticity estimates are in line with international evidence and are robust to outliers in the data.

Conclusion. Our estimates of the price elasticity of demand for cigarettes suggest that the authorities in Uganda can reduce cigarette consumption and simultaneously increase tax revenues by increasing the excise taxes on cigarettes.

Keywords: Economics; Taxation; Public Policy; Price

For peer review only

Strengths and Limitations of this study

- This paper aims at filling the evidence gap for the local impact of excise taxes on cigarette demand in an African setting with specific reference to Uganda.
- There is yet to be a published estimate of the price elasticity of demand for cigarettes in Uganda, a country where excise taxes are low.
- The paper uses the method pioneered by Angus Deaton that exploits the fact that prices of most commodities in developing countries vary across geographical space.
- A limitation is that the paper only estimates conditional price elasticities (for current cigarette smokers only) and not total price elasticities (conditional plus participation elasticities).

For peer review only

INTRODUCTION

Uganda's smoking prevalence estimates are relatively high by African standards. According to the World Health Organization (WHO), estimates for adult smoking prevalence for males and females were 17% and 3% respectively in 2016 [1]. Comparable estimates for Africa place adult male and female smoking prevalence at 17% and 2% respectively [1]. Among Ugandan youths, smoking prevalence is even higher at 19% for males and 16% for females [2]. Prevalence is relatively high in Uganda in comparison to other African countries because Uganda has, in the recent past, experienced relatively impressive economic growth with a modest increase in the real price of tobacco products. For example, Ho et al. [3] estimate that Gross National Income Per Capita increased by 107% between 1999 and 2013. Over the same period, real cigarette prices only increased by 42% [3]. Therefore, smoking prevalence and tobacco consumption in Uganda will increase in the coming years if these trends continue.

Increasing excise taxes on tobacco products is the single-most effective policy tool for reducing the demand for tobacco products [4, 5]. Unfortunately, excise taxes in Uganda are low and well below the WHO's recommended target. The percentage of the cigarette retail price that is due to the excise tax (known as the excise tax burden) was estimated at 40% in 2017 [2]. WHO recommends an excise tax burden of at least 70% of the retail price [6]. We know from several studies that cigarettes (and all other tobacco products) are generally price inelastic. Available estimates put the price elasticity of demand for cigarettes in the range of -0.20 to -0.60 [4, 5]. The implication of these estimates is that by increasing taxes on cigarettes, countries can decrease consumption and at the same time increase tax revenues.

For peer review only - http://bmjopen.bmj.com/site/about/guidelines.xhtml

To the best of our knowledge, there are no published estimates of the price elasticity of demand for cigarettes that focus specifically on Uganda. As a matter of fact, there are very few published estimates of the price elasticity of demand for cigarettes and tobacco products in Africa. The comprehensive survey of the literature on the price elasticity of demand for cigarettes by the International Agency for Research on Cancer was only able to list two sub-Sahara African countries (South Africa and Zimbabwe) as having published estimates of the price elasticity of demand [5]. These studies found price elasticity estimates ranging between -0.16 and -1.52. Using panel data techniques, Ho et al. [3] have recently provided estimates of the price elasticity of demand for cigarettes for the African continent using a sample of 36 countries. Their full-sample estimate of -0.50 is right within the range of international estimates [4, 5]. Much like the international literature, Ho et al. [3] show that there is heterogeneity in elasticity estimates by income category. Low-income African countries have, as a group, the highest elasticity estimates with high-income countries having the lowest estimates.

Even though we expect cigarettes to be price inelastic in most countries, policymakers still demand local evidence before initiating policy changes. There is, therefore, a need to generate local evidence in Africa to support civil society's efforts in encouraging governments to increase the excise tax as part of a comprehensive strategy. Often generating local evidence is constrained by the unavailability of appropriate data. The method used in this paper utilizes expenditure data, widely available on the continent, to estimate elasticities. The fact that expenditure data are widely available on the African continent means that Deaton's method holds much promise for generating local evidence on the continent. A small literature has recently emerged that uses Deaton's method

in estimating price elasticities of demand for cigarettes in Low- and Middle- Income Countries [7 – 11]. This literature's price elasticity estimates (-0.10 to -0.60) are in line with studies that use more traditional time series methods (-0.20 to -0.60). This method is yet to be applied to an African country and Uganda provides us with a unique opportunity to demonstrate the efficacy of the method.

METHOD

This paper uses the method proposed by Deaton [12] and extended in subsequent years [13 - 15] to estimate price elastcities for cigarettes in Uganda. The method uses the fact that prices of most goods in Low- and Middle-Income Countries (LMICs) are similar within narrowly defined clusters like villages but vary across clusters owing to the presence of significant transportation costs as goods move from one cluster to the next. The fact that price variation is largely induced by an external factor (transportation costs) allows one to estimate demand elasticities that are free from concerns of reverse causality or simultaneity bias. Deaton's method has an added layer of attraction for researchers working in Africa because it only uses household expenditure surveys which are available in abundance on the continent. Traditionally, the literature on tobacco demand has estimated price elasticities using aggregate time series data on cigarette consumption and prices. This partly explains why there are only a handful of countries on the continent with estimates of the price elasticity of demand: many African countries do not maintain time series data of a sufficient time length to allow for a proper estimate of elasticities. On the other hand, many African countries often conduct household expenditure surveys every so often for the purposes of constructing, among other things, Consumer Price Indices (CPIs).

For peer review only

Deaton's method proceeds in a series of steps. First, the researcher extracts unit values (a proxy for prices) from the survey data at the household level. This is done by dividing total expenditure on cigarettes by the quantity demanded of cigarettes or algebraically as

$$\upsilon_{ic} \equiv \frac{x_{ic}}{q_{ic}} \qquad (1)$$

where υ_{ic}, x_{ic} and q_{ic} are respectively the unit value, expenditure and quantity of cigarettes in household i located in cluster c. Deaton uses unit values as opposed to actual prices because household expenditure surveys rarely collect information on the prices faced by individual households. However, unit values are not the same thing as prices. For one thing, unit values hide a great degree of quality heterogeneity whereas the classic treatment of demand concerns itself with homogeneous goods. With quality heterogeneity, households may respond to a price increase by shifting to a lower "quality" brand of cigarettes with a small decline in quantity. Deaton refers to this as "quality shading". With quality shading, the price elasticity of demand will be overestimated. Secondly, unit values are not the same as prices because of measurement error. Households are unlikely to correctly recall the amount of money spent on cigarettes and/or the quantity consumed. In some cases, the survey enumerator might incorrectly capture this information. In such a case, (1) would result in a wrong price even if cigarettes were a homogeneous product (i.e. cigarettes were not susceptible to quality shading). The presence of measurement error will bias the estimate of the price elasticity of demand. Fortunately Deaton has

For peer review only

proposed formulae that allow the researcher to correct their final elasticity estimates of quality shading and measurement error (see below).

The second step in Deaton's method involves checking whether the main identifying assumption, that prices (unit values) vary spatially, holds for the unit values obtained above. This is done by using Analysis of Variance (ANOVA) to divide the total variation in unit values into "within cluster variation" and "between cluster variation". A large F statistic for the ANOVA exercise leads to the conclusion that unit values vary across space. In a third step, one estimates within cluster regressions of the form:

$$lnv_{ic} = \lambda + \beta lnx_{ic} + \boldsymbol{\gamma Z_{ic}} + \psi ln\pi_c + e_{ic} \qquad (2) \text{ and}$$

$$w_{ic} = \alpha + \varepsilon lnx_{ic} + \boldsymbol{\delta Z_{ic}} + \theta ln\pi_c + (FE_c + u_{ic}) \qquad (3)$$

w_{ic} represents the share of cigarette expenditure in total household expenditure for household i in cluster c and lnv_{ic} is the log of the unit value, derived according to identity (1) for household i in cluster c. lnx_{ic} is the log of total household expenditure over the relevant reference period. $\boldsymbol{Z_{ic}}$ is a vector of household specific characteristics including household size, household gender composition, gender of the household head, proportion of adults in the household and years of schooling of the household head. Other variables in $\boldsymbol{Z_{ic}}$ are the age of the household head and a dummy variable for whether the household head is formally employed or not. FE_c is a cluster fixed effect. u_{ic} and e_{ic} are the standard regression error terms. $ln\pi_c$ are the unobserved prices and

For peer review only

consequently, equations (2) and (3) are estimated without them. The coefficients on the price terms can, however, be recovered (see below).

Equation (2), referred to as the "unit value" equation, allows us to check for the presence of quality effects in the unit value data. A positive and statistically significant relationship between household expenditure and unit values, after accounting for household characteristics, would suggest the presence of quality effects. That is, richer households report higher unit values primarily because they are buying cigarettes of a higher quality. Knowing the pattern of the quality effects (i.e. the magnitude of β), allows us to correct our final price elasticity estimates for quality shading. It is also important to note that some portion of the variation in unit values comes from these choices in quality that households are making. This variation is also exploited below in estimating our elasticities. Equation (3), on the other hand, is a standard demand equation where the cigarette share is expressed as a function of household income (proxied by household expenditure), household characteristics and prices. The cluster fixed effects allow us to control for cluster-level tastes and preferences. Similar tastes and preferences are to be expected for narrowly constructed clusters such as villages. Equations (2) and (3) also contain useful information about measurement error at the household level. The magnitude of the errors are captured by e_{ic} and u_{ic}, the regression error terms. The relationship between the two errors (as captured by, say, the covariance) is useful in correcting the final price elasticity estimates for measurement error (see more below).

For peer review only

The fourth step involves stripping the household-level demand and unit values of the effects of household expenditure and household characteristics and then averaging across clusters. This step requires the following equations:

$$\widehat{y_c^1} = \frac{1}{n_c}\sum_{i=1}^{n_c}(lnv_{ic} - \hat{\beta}lnx_{ic} - \hat{\boldsymbol{\gamma}}\boldsymbol{Z}_{ic}) \qquad (4) \text{ and}$$

$$\widehat{y_c^2} = \frac{1}{n_c}\sum_{i=1}^{n_c}(w_{ic} - \hat{\varepsilon}lnx_{ic} - \hat{\boldsymbol{\delta}}\boldsymbol{Z}_{ic}) \qquad (5),$$

where n_c is the number of households in cluster c. $\widehat{y_c^1}$ and $\widehat{y_c^2}$ are the estimates of, respectively, cluster average unit value and cluster average demand after removing the effects of household expenditure and household characteristics (notice that $\widehat{y_c^1}$ and $\widehat{y_c^2}$ do not have the i subscript because they represent cluster averages). Recall that our identifying assumption is that prices vary at the cluster level (i.e. between clusters and not within clusters). Given this identifying assumption, price elasticities of demand can only be obtained by seeing how cluster-level demand responds to changes in cluster-level prices. This leads to the fifth step which involves regressing cluster-level demand, $\widehat{y_c^2}$, on cluster-level unit values, $\widehat{y_c^1}$. The coefficient on $\widehat{y_c^1}$ in such a regression can alternatively be obtained by dividing the covariance between $\widehat{y_c^2}$ and $\widehat{y_c^1}$ by the variance of $\widehat{y_c^1}$. That is $\hat{\phi}$, the estimate of the coefficient on y_c^1, is obtained by

$$\hat{\phi} = \frac{Cov\left(\widehat{y_c^2}, \widehat{y_c^1}\right) - \frac{\widehat{\sigma^{12}}}{n_c}}{Var\left(\widehat{y_c^1}\right) - \frac{\widehat{\sigma^{11}}}{n_c^+}} \qquad (6),$$

For peer review only

where n_c^+ is the number of households in a village reporting positive expenditures on tobacco and n_c is the number of households in a village; $\widehat{\sigma^{12}}$ is the estimate of the covariance of the errors in equations (2) and (3); $\widehat{\sigma^{11}}$ is the variance of the errors in equation (2). These are included in equation (6) to adjust our estimates for measurement error.

The sixth and final step in Deaton's method applies quality correction formulas in obtaining the estimate of the price elasticity of demand, $\widehat{\varepsilon_P}$, as follows:

$$\widehat{\varepsilon_P} = \left(\frac{\hat{\theta}}{\overline{\overline{w}}}\right) - \hat{\psi} \qquad (7),$$

where $\overline{w}$ is the average share of total household expenditure dedicated to cigarettes in the sample. $\hat{\psi}$ and $\hat{\theta}$, the estimates of the coefficients on the unobserved price terms in equations (2) and (3) respectively, are recovered as follows:

$$\hat{\psi} = 1 - \frac{\hat{\beta}(\overline{w} - \hat{\theta})}{\hat{\varepsilon} + \overline{w}} \qquad (8) \text{ and}$$

$$\hat{\theta} = \frac{\hat{\phi}}{1 + (\overline{w} - \hat{\phi})\hat{\zeta}} \qquad (9) \text{ with}$$

$$\hat{\zeta} = \frac{\hat{\beta}}{\hat{\varepsilon} + \overline{w}(1 - \hat{\beta})} \qquad (10).$$

$\hat{\beta}$ is the estimate of the coefficient on total household expenditure in equation (2), the within-cluster unit value equation, and $\hat{\varepsilon}$ is the estimate of the coefficient on total household expenditure in equation (3), the within-cluster demand equation. $\hat{\phi}$ is the estimate of the coefficient of a regression of cluster-level demand on cluster-level unit value (from equation 6).

Deaton also proposes the following formula for obtaining the estimate of the expenditure elasticity of demand, $\widehat{\varepsilon_I}$:

$$\widehat{\varepsilon_I} = 1 + \left(\frac{\hat{\varepsilon}}{\overline{w}}\right) - \hat{\beta} \qquad (3.11).$$

Data

The data come from the 2005 and 2009 editions of Uganda's National Panel Survey (UNPS), a nationally representative survey. The UNPS is conducted by the Uganda Bureau of Statistics with technical assistance from the World Bank. Households that were surveyed in 2005 were revisited in 2009. Additional panel studies have been conducted since 2009. This paper does not use data from latter rounds because these surveys did not collect sufficiently detailed information on the quantities of cigarettes purchased by households. Specifically, information on whether the unit of purchase was a stick, packet or bundle was missing from the latter rounds of the survey. Deaton's

For peer review only

method requires that the units for the quantity variable be the same. Further, we do not exploit the panel characteristics of the paper but instead treat 2005 and 2009 as separate cross-sections.

The UNPS collects an exhaustive list of information on the socio-economic characteristics of households. This includes information on household expenditure patterns, household composition patterns, and the occupational and educational status of household members. In the household expenditure module of the survey, which is this paper's primary interest, households are required to recall expenditure on various goods and services. The recall period ranges from 7 days for cigarettes to one year for household expenditures on education, health and clothing to name a few. We convert all expenditures into weekly expenditures.

The expenditure module collects information on the quantity and total amount paid for cigarettes over the last 7 days. The quantities are recorded either as sticks or packets. We convert sticks into packets by dividing them by 20, which is the number of cigarettes in a pack in Uganda. Another unfortunate aspect of the Uganda National Panel Survey is that it does not distinguish between zero expenditures and expenditure information that is simply missing. In other words, a household's information on cigarette expenditure is left out of the survey if (1) the household reported zero expenditure on cigarettes in the previous week or (2) the household was never asked this question or (3) the respondent did not know the answer to the question. Notice that case (1) coincides with a scenario where the household consumes cigarettes but did not do so in the previous week because, say, the price was high (i.e. a corner solution). It also coincides with a scenario where the household does not consume any cigarettes at all. On the other hand, cases (2) and (3) are scenarios where information is simply missing. Because the survey is not explicit about

For peer review only

distinguishing zero expenditures from missing expenditures, we choose to only use households that reported positive cigarette expenditures in estimating the price elasticity of demand. In other words, we estimate conditional price elasticities of demand. To allow for comparisons across years, we express all unit values and total expenditures in terms of 2010 prices using Uganda's Consumer Price Index (CPI) [16].

Table 1 reports summary statistics for the relevant variables from the data.

Table 1: Summary Statistics from the Uganda National Panel Survey

Variable	2005	2009
Percentage of households with positive cigarette expenditure	9%	7%
Average cigarette share in total household expenditure	8.86%	7.67%
Real average unit value per pack	1,952.11	1,056.26
Average unit value per pack (USD equivalent)	0.83	0.45
Average weekly quantity of cigarettes (in packs)	1.45	1.55
Ave. real h/hold expenditure (last 7 days in Ugandan Shillings)	34,598.08	32,784.07
Ave. household expenditure (last 7 days USD equivalent)	14.72	13.95
Average household size	5.58	6.33
Average age of household head in years	41.13	43.73
Average proportion of male heads	86.81%	90.17%
Average proportion of adults	54.58%	46.88%
Average proportion of males in household	53.27%	52.00%
Average years of schooling of household head	6.55	5.81
Average proportion of heads with some employment	89.74%	84.43%
Total number of clusters	322	318
Total number of effective clusters	178	121
Total number of households	274	173
Average number of households per cluster	1.54	1.43

Notes: Summary statistics for the relevant variables from the 2005 and 2009 Uganda National Panel Survey (UNHS). Adults are those household members who are 18 years or older.

For peer review only

8% of the sample of households from 2005 reported positive expenditures on cigarettes in the last 7 days. For 2009, this number was 7%. The share of the weekly budget allocated to cigarette expenditure was, on average, 8.86% in 2005 and 7.67% in 2009. Although Uganda's cigarette expenditure share is relatively high, it falls within the range identified by John [17] particularly for LMICs. The reported average unit value per pack in 2010 Ugandan Shillings was 1,952 (83 US cents) in 2005 and 1,056 (45 US cents) in 2009 [18]. To the extent that unit values are a good proxy of actual prices, then the foregoing would suggest that cigarettes became cheaper, in real terms, by about 46% over the four year period in question. The average weekly household expenditure expressed in 2010 Uganda shillings was 34,598 (USD15) in 2005 and declined slightly by 5% to 32,784 (USD14) in 2009. The rest of the summary statistics in Table 1 mainly pertain to the ***Z*** vector of control variables used in the regressions (see equations 2 and 3 above).

Patients and public involvement

Patients and the public were not involved in this study.

RESULTS

The main identifying assumption behind Deaton's method is that prices vary across geographical space. The validity of this assumption can be tested using Analysis of Variance (ANOVA) techniques. We report the results of the ANOVA exercise in Table 2. According to the results of the ANOVA procedure, most of the variation in unit values takes place between clusters. The R-squared, which measures the proportion of total variation in unit values between clusters, is at least 70% in all three data specifications. The F statistics associated with a null hypothesis of no spatial variation are all large with the exception of the 2009 sample. Even with the 2009 sample, the R-

For peer review only

squared is 72%. In large part, the assumption of spatially varying prices is confirmed by the data. John [7, 8] also finds that cluster effects explain at least 70% of the variation in unit values in India.

Table 2: Testing the Spatial Variation Hypothesis

2005 sample				2009 sample			
F statistic	p value	R-squared	n	F statistic	p value	R-squared	n
1.29	0.08	0.70	274	1.12	0.33	0.72	173

Notes: The F statistic and the p-value are associated with the null hypothesis of no spatial variation in unit values. The hypothesis is rejected in the 2005 sample but not in the 2009 sample. The R-squared measures the proportion of variation in prices taking place between clusters. n is the total number of households.

Tables 3 and 4 show the results of running the unit value and budget share regressions (equations 2 and 3) on the two samples. The results for the unit value regression are reported in Table 3. The results show that quality effects are present in the data: households with higher household expenditure tend to report higher unit values controlling for other household characteristics and controlling for year effects in the pooled sample. Reported unit values rise by between 1% and 2% for every 10% increase in household expenditure. These so-called expenditure elasticities of quality compare favourably with those estimated for cigarettes by John [7, 8], Eozenou and Fishburn [9], Guindon et al. [10] and Chen and Xing [11].

Table 3: Results of the unit value regression (equation 2)

Variables	(2005) lnv	(2009) Lnv
lnx	0.234***	0.115**
	(0.051)	(0.048)
Size	-0.042	-0.010
	(0.124)	(0.119)
Adults	-0.203	0.159
	(0.295)	(0.300)
Males	0.261	0.131
	(0.216)	(0.223)
Education	-0.143*	0.108
	(0.080)	(0.074)
Age	-0.015	-0.409**
	(0.153)	(0.166)
Gender	0.217	0.218
	(0.163)	(0.183)
Work	-0.144	0.101
	(0.141)	(0.118)
Year		
Constant	4.957***	6.602***
	(0.692)	(0.739)
No. of households	233	147
R-squared	0.115	0.126

Notes: Results of the regression of the log of unit value (lnv) on the log of household expenditure (lnx) and other household characteristics (see equation 2). Household size (Size), education of household head (Education) and age of household head (Age) are in natural logarithms. Adults refers to the proportion of adults in a household and adults are defined as aged 18 years or older. Males is the proportion of males in a household. Gender is a dummy variable which takes on the value of 1 if the household head is male and zero if they are female. Work is a dummy variable which takes on the value of 1 if the household head is employed and zero otherwise.. Standard errors in parentheses. *** p<0.01, ** p<0.05, * p<0.1.

For peer review only

Table 4 reports the results of running the budget share regression (equation 3) on household expenditure alongside other household characteristics and cluster fixed-effects. In Table 4, there is a negative and statistically significant relationship between household expenditure and the share of the household budget allocated to cigarettes. The cigarette budget share tends to fall as household expenditure rises. The cluster fixed effects, whose results are not presented in Table 4 for purposes of space, are all jointly statistically significant at the 1% level for the 2005 and at the 10% level for the 2009 sample. This means that unobserved but fixed factors at the cluster level (tastes, preferences, etc…) matter for cigarette demand.

For peer review only

Table 4: Results of the budget share regression (equation 3)

Variables	(2005) w	(2009) W
lnx	-0.056***	-0.065***
	(0.017)	(0.023)
Size	0.002	0.039
	(0.031)	(0.043)
Adults	0.008	0.092
	(0.072)	(0.103)
Males	0.013	0.010
	(0.059)	(0.068)
Education	-0.001	-0.012
	(0.020)	(0.025)
Age	0.028	-0.077
	(0.044)	(0.072)
Gender	-0.038	-0.108*
	(0.037)	(0.056)
Work	0.037	0.058
	(0.037)	(0.039)
Year		
Constant	0.533***	0.963***
	(0.193)	(0.292)
No. of households	233	147
R-squared	0.866	0.909

Notes: Results of the regression of the cigarette budget share (w) on the log of household expenditure (lnx) and other household characteristics (see equation 3). Household size (Size), education of household head (Education) and age of household head (Age) are in natural logarithms. Adults refers to the proportion of adults in a household and adults are defined as aged 18 years or older. Males is the proportion of males in a household. Gender is a dummy variable which takes on the value of 1 if the household head is male and zero if they are female. Work is a dummy variable which takes on the value of 1 if the household head is employed and zero otherwise. Standard errors in parentheses. *** p<0.01, ** p<0.05, * p<0.1. Cluster fixed effects are suppressed for space reasons but are jointly statistically significant at the 1% level for the 2005 sample and at 10% for the 2009 sample.

Table 5 presents our estimates of the price elasticity of demand for cigarettes in Uganda. The estimates are obtained using the information in Tables 3 and 4 alongside the formulae in equations 4 to 10 above. Standard errors (in parenthesis) are obtained by the method of the Bootstrap.

Table 5: Estimates of the price elasticity of demand for cigarettes in Uganda

	**(2005)**	**(2009)**
$\hat{\varepsilon}_P$	-0.326***<br>[0.021]<br>(-0.368 , -0.284)	-0.258***<br>[0.011]<br>(-0.280 , -0.235)
No. of households	233	147
No. of clusters	184	130

Notes: Estimates of the price elasticity of demand for cigarettes in Uganda for the 2005, 2009 and pooled samples. Bootstrapped standard errors are in square brackets. 95% confidence intervals are in parentheses. *** p<0.01, ** p<0.05, * p<0.1.

The estimates in Table 5 show that cigarettes are price inelastic in Uganda. The estimates for the price elasticity of demand for the two samples are all statistically significant at the 1% level. For 2005, the price elasticity is estimated at -0.33 while for the 2009 sample the estimate is at -0.26. Taken together, these results imply that the demand for cigarettes in Uganda is expected to decline by about 3% for every 10% rise in prices. These results fall within the range of estimates for studies that use Deaton's method (-0.10 to -0.60) and studies using aggregate time series data (-0.20 to -0.60).

For peer review only

Table 6 presents estimates of the expenditure elasticity of demand using equation (11) and coefficient estimates from Tables 3 and 4. The estimates in Table 6 are imprecisely estimated and are, therefore, statistically insignificant.

Table 6: Estimates of expenditure elasticities of demand for cigarettes in Uganda

	**(2005)**	**(2009)**
$\hat{\varepsilon}_I$	0.132<br>[0.338]<br>(-0.531 , 0.796)	0.043<br>[0.539]<br>(-1.014 , 1.100)
No. of households	233	147

Notes: Estimates of the expenditure elasticity of demand for cigarettes in Uganda for the 2005 and 2009 samples. Bootstrapped standard errors are in square brackets. 95% confidence intervals are in parentheses. Since the expenditure elasticity of demand is estimated at the household level (see equation 11), we only report the number of households.

For peer review only

Robustness

The estimates of the price elasticity of demand for cigarettes presented above are likely to be influenced by extreme unit values given that the sample sizes are relatively small. To check the robustness of our estimates, we follow Guindon et al. [10] and exclude unit values that are greater than 2.5 standard deviations and 5 standard deviations from their respective means. (Given the similarity of the results in Tables 5 and the better performance of the 2005 sample in the ANOVA exercise, we only perform the robustness test on the 2005 sample).

Table 7 contains the estimates of the price elasticity of demand for cigarettes for the robustness tests. We have also included in Table 7, estimates for the 2005 sample taken from Table 5. The second column (column 2) excludes unit values that deviate by more than 5 standard deviations from the mean while the third column (column 3), being more stringent, excludes unit values that

deviate by more than 2.5 standard deviations from the mean. The price elasticity estimates in columns 2 and 3 are statistically significant at the 1% level and 5% level respectively and are similar to the estimate from the 2005 sample taken from Table 5.

Table 7: Estimates of price elasticities of demand for cigarettes in Uganda (Robustness check)

	(2005 Full sample)	(2)	(3)
$\hat{\varepsilon}_P$	-0.326***	-0.351***	-0.271**
	[0.021]	[0.054]	[0.120]
	(-0.368, -0.284)	(-0.458 , -0.246)	(-0.526 , -0.017)
No. of households	233	231	227
No. of clusters	184	176	175

Notes: Estimates of the price elasticity of demand for cigarettes in Uganda for the 2005 sample and for the 2005 sample with extreme unit values excluded. Column 1 (2005 sample) includes all unit values. In column 2, all unit values that are equal to or greater than 5 standard deviations from the mean are excluded. In column 3, all unit values that are equal to or greater than 2.5 standard deviations from the mean are excluded. Bootstrapped standard errors are in square brackets. 95% confidence intervals are in parentheses. *** p<0.01, ** p<0.05, * p<0.1.

DISCUSSION

This paper had two objectives in mind. First, to provide the first ever estimates of the price elasticity of demand for cigarettes in Uganda, a country where prevalence and consumption are expected to increase in the future. The experience in other African countries, particularly South Africa [19], shows that consistently increasing excise taxes can play a significant role in reducing consumption. The evidence generated in this paper will be useful for policy makers and civil society actors involved in tobacco control efforts in Uganda. The second aim for the paper was to demonstrate the efficacy of using Deaton's method in an African setting where the unavailability of appropriate data has in the past prevented the estimation of price elasticities. Deaton's method

For peer review only

relies on expenditure data which is widely available across the African continent. This, alongside with the fact that our estimates for Uganda are in line with international evidence [3 – 5] and in line with studies using Deaton's method [7 -11], will hopefully encourage other researchers working on the continent to consider using this method for generating local evidence.

Our study focussed on cigarettes because the first two rounds of the UNPS did not collect detailed enough information on other tobacco products to allow for the estimation of their elasticities using Deaton's method. Further, we were only able to estimate conditional elasticities because the UNPS does not distinguish between households reporting zero expenditure on cigarettes and those whose information is missing. Therefore, the total price response of cigarettes in Uganda is likely to be twice our estimated conditional elasticity of -0.30 given the empirical finding that the total elasticity is often double the magnitude of the conditional elasticity [5]. This is similar to the finding in Ho et al. [3] where the [total] price elasticity of low-income African countries, in which Uganda is categorized, is estimated at -0.56.

Given that our study only focussed on cigarettes in Uganda, future research should aim at estimating price elasticities for other tobacco products and cross-price elasticities between cigarettes and other types of tobacco products . Cross price elasticities are important to know given the fact that levying taxes on cigarettes might not only affect cigarette demand, but could also increase the demand for other types of tobacco products. This, however, requires that the statistical authorities in Uganda consider collecting expenditure information on other types of tobacco products.

For peer review only

Contributors:

GC did the analysis and wrote-up the paper together with CvW.

Funding:

This work was supported by funding from the Bill and Melinda Gates Foundation.

Competing Interests: None.

Provenance and peer review: Not commissioned; externally peer reviewed

Data sharing statement: All datasets are publicly available.

For peer review only

References

1. World Health Organization. Global Health Observation Data. 2018. Available at: http://www.who.int/gho/tobacco/use/en/ (Accessed June 2018)
2. World Health Organization. Report on the Global Tobacco Epidemic. Geneva: World Health Organization 2017
3. Ho L-M, Schafferer C, et al. The effect of cigarette price increases on cigarette consumption, tax revenue, and smoking-related death in Africa from 1999 to 2013. International Journal of Public Health 2017; 8: 899 – 909
4. Chaloupka F, Warner KE. The economics of smoking. In: Newhouse J, Cutler D, eds. Handbook of Health Economics. Amsterdam: North Holland 2000: 1539 – 1567
5. International Agency for Research on Cancer. Handbook on the effectiveness of tax and price policies for tobacco control. Geneva: World Health Organization 2011
6. World Health Organization.Technical Manual on Tax Administration. Geneva: World Health Organization 2010
7. John RM. Price elasticity estimates for tobacco and other addictive goods in India. Indira Gandhi Institute of Development Research 2005; Working Paper Number WP-2005-003
8. John RM. Price elasticity estimates for tobacco products in India. Health Policy and Planning 2008; 23: 200 – 209
9. Eozenou P, Fishburn B. Price elasticity estimates for cigarette demand in Vietnam. Development and Policies Research Center 2009; Working Paper Number 5
10. Guindon EG, Nandi A, et al. Socioeconomic differences in the impact of smoking tobacco and alcohol prices on smoking in India. National Bureau of Economics Research 2011; Working Paper Number 17580

For peer review only

11. Chen Y, Xing W. Quantity, quality and regional price variation of cigarettes: Demand analysis based on a household survey in China. China Economic Review 2011; 22: 221 – 232

12. Deaton A. Analysis of Household Surveys. Baltimore: Johns Hopkins Press 1997

13. Deaton A. Quality, Quantity, and Spatial Variation of Price. American Economic Review 1988; 78: 418 – 430

14. Deaton A. Household Survey Data and Pricing Policies in Developing Countries. World Bank Economic Review 1989; 3: 183 – 210

15. Deaton A. Price elasticities from Survey Data: Extensions and Indonesian Results. Journal of Econometrics 1990; 44: 281 – 304

16. We obtain the CPI from the World Development Indicators provided by the World Bank. Available here: http://data.worldbank.org/data-catalog/world-development-indicators

17. John RM. Crowding out effect of tobacco expenditure and its implications on household resource allocation in India. Social Science and Medicine 2008; 66: 1356 – 1367

18. We convert 2010 Uganda shillings into US dollars by using the 2010 end of year exchange rate between the shilling and the dollar available from www.oanda.com.

19. Chelwa G, Van Walbeek C, Blecher E. Evaluating South Africa's tobacco control policy using a synthetic control method. Tobacco Control 2017; 26: 509 – 517

For peer review only
