# Supplementary material for: Does cigarette demand respond to price increases in Uganda? Price elasticity estimates using the Uganda National Panel Survey and Deaton’s method
Source: BMJ Open. 2019 Mar 20;9(3):e026150. doi: 10.1136/bmjopen-2018-026150 (PMC6475351; doi:10.1136/bmjopen-2018-026150)
Supplement: Reviewer comments [file bmjopen-2018-026150.reviewer_comments.pdf]

PEER REVIEW HISTORY

BMJ Open publishes all reviews undertaken for accepted manuscripts. Reviewers are asked to complete a checklist review form (**http://bmjopen.bmj.com/site/about/resources/checklist.pdf)** and are provided with free text boxes to elaborate on their assessment. These free text comments are reproduced below.

ARTICLE DETAILS

**TITLE (PROVISIONAL)**	Does Cigarette Demand respond to price increases in Uganda? Price Elasticity Estimates using the Uganda National Panel Survey and Deaton's Method
**AUTHORS**	Chelwa, Grieve; Walbeek, Corne

VERSION 1 - REVIEW

**REVIEWER**	Guillermo Paraje<br>Universidad Adolfo Ibáñez Chile<br>I am the Principal Investigator for Chile of a project where Dr Corne Van Wallbeck is also a Principal Investigator (for South Africa)
**REVIEW RETURNED**	07-Oct-2018

**GENERAL COMMENTS**	Overall, the manuscript is very well-written, clear and with well-defined objectives. The topic addressed in the article is relevant to Uganda but also to other developing countries, especially those of Sub-Saharan Africa, where very little research is conducted on tobacco control topics. As the authors point out, the main findings are in line with similar studies conducted for other countries.<br><br>I a few comments that may help improve the, otherwise, well-written manuscript. Most of them deal with the methods. The authors use a linear variation of Deaton's AIDS, which is fine. However, I believe there might be some confusion with some aspects of the method. First, in page 5 of the copy I got, they say: "The fact that price variation is largely induced by an external factor (transportation costs) allows one to estimate demand elasticities that are free from concerns of reverse causality or simultaneity bias." I believe this is partly true (and a bit misleading). As I understand it, Deaton's AIDS exploits price variation that is induced by external factors, but it also exploits variation that is caused by the quality shading. Households from different villages face a different set of prices (because of external factors, such as transportation costs), but households from the same village may choose goods with different prices (proxied by unit values). I believe AIDS exploits both variations to identify demand. According to the authors results, a good deal of such variation is within clusters. I believe the authors can do a better job at explaining this and how this is incorporated in the results.<br><br>In page 6, the authors say that with quality shading price elasticity of demand will be overestimated. Is that so? Let's assume for instance that a change in price of a cigarette expensive brand, widely smoked, leads to the down-trading to a cheaper brand but the quantity of cigarettes smoked remains the same. Basically, that would mean that the estimated elasticity is close to zero, though it has been a change in behaviour. Is elasticity overestimated or underestimated in such a case? May be the authors can answer this

question and use this example to make this point clearer.

Just after that claim, the authors say that unit values are not the same as prices because of measurement errors (by the way, equation (1) is not an equation: it's an identity). I do not think this is correct. Unit values are different from prices even if everything is recorded perfectly. The main difference between them is that households are price-takers but can choose unit values of the goods they choose to buy. Prices are exogenous to households' choices; unit values are endogenous. That's the main difference (Deaton specifically states this) and, unless I am wrong, the authors should change this. They say: "Households are unlikely to correctly recall the amount of money spent on cigarettes and/or the quantity consumed. In some cases, the survey enumerator might incorrectly capture this information." The same could be said about price recording. One could ask about quantities and prices and there would be measurement errors.

On page 8, they say: "Knowing the pattern of the quality effects (i.e. the magnitude of [illegible]), allows us to correct our final price elasticity estimates". Usually, parameters associated with "quality" are associated with the income elasticity of demand. How is [illegible] associated with price elasticity? It's not obvious at all, from the equation of price elasticity (7). Please, clarify this point.

In addition, why there is no estimation of the income elasticity of demand? I believe it would be very important to have this estimate or to justify why is missing.

On page 8, further below, they claim: "Because of the assumption that prices are fixed within clusters and the fact that we do not have price data, prices are proxied by cluster fixed effects". Again, my understanding of Deaton's AIDS tells me this interpretation is wrong. Prices are proxied by unit values and household characteristics. Fixed effects capture tastes, preferences and idiosyncratic factors, as they authors claim right after that sentence. If fixed effects capture prices, tastes, preferences, etc; how is it possible to untangle the price from the other variables to estimate elasticity? I might be wrong, but anyway this needs to be explained better.

Another question is: given that they cannot estimate a "participation elasticity" by using households that choose to buy cigarettes and those who choose not to buy, is there any self-selection bias in the sample? Why not using, for instance, Heckman's method to correct for that? Please, clarify why this is not done.

Table 1 could also inform the number of clusters (or the proportion of them out of the total) that have households used in the final estimation. If there is a large proportion of them that are not used, the authors should discuss the implications of this. In addition, it would be nice to understand why the average proportion of households per cluster is so low. Usually clusters are a set of blocks or small neighbourhoods or even villages. Less than two households per cluster seems a very small amount. Is this correct? Is there any implication of this in the results (see comment just below)?

Table 5 shows the results with bootstrapped standard errors. How many times was the sample bootstrapped? Was the bootstrap made for the whole sample or was made considering some clustering of households? Any implication of the latter?

**REVIEWER**	Nigar Nargis<br>American Cancer Society, United States
**REVIEW RETURNED**	15-Oct-2018

**GENERAL COMMENTS**	This is a well written paper and makes important contribution to the economics of cigarette smoking in the context of Sub-Saharan Africa. It uses a household panel survey to estimate price elasticity of conditional cigarette demand in Uganda.<br><br>While the study makes the best use of available data in applying Deaton's model, authors need to make further clarifications to the methods of data analysis and recognize the limitations of the study more clearly.<br><br>1. Table 1 shows average household expenditure decreases in real terms. Is it consistent with overall economic growth in the country?<br><br>2. There are less than two households per cluster on average. What is the range of the number of households by cluster? I am guessing that some clusters may have a single household. It can cause a limitation in using spatial variation in price for the identification of the effect of price changes. Because in these cases the cluster unit value is essentially the unit value of a household, which can hardly represent a full cluster. In fact, the finding in ANOVA that about 30% residual variation in unit value is due to within cluster variation might be an underestimate because of the representation of some clusters with single households. For these clusters, 100% variation comes from between cluster variation and cluster fixed effects cannot be estimated in equation 3.<br><br>3. What is the retention rate of the panel of households in 2009? The numbers of households and clusters reduced significantly between 2005 and 2009. Can it create any systematic bias in the analysis? Does it have to do with the lack of spatial variation in prices observed in the 2009 sample in the ANOVA exercise? What other factor variables were used in ANOVA?<br><br>4. How large are the samples of pack purchases and stick purchases? Authors can test if pack versus stick purchases can contribute to the variation in prices by using it as a factor variable in ANOVA.<br><br>5. In Table 3, the coefficient of Year indicates that unit values were lower in 2005 than in 2009 according to the description of the Year dummy variable in the Notes: Year takes on the value of 1 if the household was surveyed in 2005 and zero if surveyed in 2009. In contrast, the average price level shown in Table 1 was higher in 2005 than in 2009. Please double check if the base year used in the regression was 2005.<br><br>6. Did you try to estimate the model using panel data regression on pooled dataset that would control for household level fixed effects? It would be worth comparing the results with the pooled regression estimates. Please explain the merit of using pooled OLS instead of fixed effects model.

	7. In the pooled regression, the error terms of the same household are expected to be correlated. Did you correct standard errors of estimates for the correlation of repeated observations?

**REVIEWER**	Christian Schafferer<br>Department of International Tradeb Overseas Chinese University Taiwan
**REVIEW RETURNED**	17-Oct-2018

**GENERAL COMMENTS**	This paper provides estimates of price elasticity of demand for cigarette consumption in Uganda. Publication certainly is in the interest of the scientific community: 1) Studies on price elasticity estimates for Uganda are rare. 2) The authors apply an alternative estimation method that may deliver more accurate results for African countries. The paper thus certainly deserves attention. However, several points should be addressed before publication.<br><br>Abstract/Strengths and Limitations<br>P3/7: I am not sure whether it is important to mention that Angus Deaton received the Nobel Prize.<br>P3/14-17: Considering only cigarette demand is quite common, and in most cases do not constitute a major limitation unless cigarette use is rare in comparison to other tobacco products. If so, this should be mentioned in the introduction/discussion section of the paper.<br><br>Introduction<br>The introduction could cover more details regarding the smoking prevalence in African countries, price elasticity figures and about the study itself. That is, a literature review is missing. Why is smoking prevalence higher than in other African countries? How about cigarette prices/availability?<br>P4/28-47 Elasticity estimates: Which countries/geographic areas are covered here? Any difference between developed and less-developed states? Why do estimates differ? Because of different methodologies?<br>P4/52-P5/10: Although studies are rare for Africa (Uganda), there is some (comparative) literature (e.g. Li-Ming Ho et. al, Int J Public Health (2017) 62: 899). What estimates have been previously reported for African countries? Do we expect Uganda do be different? What is so special about Uganda (or perhaps for the whole of Africa) so that the application of Deaton's methodology is necessary/more promising. I am aware that the authors state several reasons in the Method section of the paper, but it would be beneficial to the reader to get an overall idea about what the paper has to offer in the Introduction section. So far, this is not mentioned clearly enough in the Introduction. Perhaps some of the explanations in the Method section can be moved to/highlighted in the Introduction.<br>Perhaps the authors could also mention whether their methodology has previously been applied in other studies on cigarette price elasticity. If so, what are the results? Any positive experience?<br>Method<br>This section of the paper is quite extensive. Perhaps this section should be used to focus on the technical aspects of the methodology, whereas shortcomings etc. could be mentioned/discussed in the Introduction/Discussion section of the

	paper.<br>For example, p.26/19-27 dealing with recently emerged literature and its results could be moved to the Discussion section.<br>Moreover, some efforts could be made to improve the 'flow' of the paper.<br>Discussion<br>This section needs greater attention. It looks more like a summary of key features/results of the study. It lacks a discussion about the problem the study endeavours to solve. Perhaps it should discuss the problem of increased smoking prevalence in Uganda and what role taxation could play in reducing cigarette use by referring to the experience of other African countries. Furthermore, it should probably discuss the question of why the applied methodology was chosen and whether expectations were met. Do the authors recommend the future application of the Deaton method? How might such results differ from other methodologies? Again, this may be done by including previously published studies.<br>How effective may taxation be in Uganda considering socio-political and institutional circumstances?<br>Moreover, apart from discussing the limitations of the study, it may be helpful to the reader to understand how future research on cigarette price elasticity could be improved.

VERSION 1 – AUTHOR RESPONSE

Reviewer: 1
Reviewer Name: Guillermo Paraje
Institution and Country: Universidad Adolfo Ibáñez, Chile
Please state any competing interests or state 'None declared': I am the Principal Investigator for Chile of a project where Dr Corne Van Wallbeck is also a Principal Investigator (for South Africa)

Please leave your comments for the authors below
See attached file

Overall, the manuscript is very well-written, clear and with well-defined objectives. The topic addressed in the article is relevant to Uganda but also to other developing countries, especially those of Sub-Saharan Africa, where very little research is conducted on tobacco control topics. As the authors point out, the main findings are in line with similar studies conducted for other countries.

1. I have a few comments that may help improve the, otherwise, well-written manuscript. Most of them deal with the methods. The authors use a linear variation of Deaton's AIDS, which is fine. However, I believe there might be some confusion with some aspects of the method. First, in page 5 of the copy I got, they say: "The fact that price variation is largely induced by an external factor (transportation costs) allows one to estimate demand elasticities that are free from concerns of reverse causality or simultaneity bias." I believe this is partly true (and a bit misleading). As I understand it, Deaton's AIDS exploits price variation that is induced by external factors, but it also exploits variation that is caused by the quality shading. Households from different villages face a different set of prices (because of external factors, such as transportation costs), but households from the same village may choose goods with different prices (proxied by unit values). I believe AIDS exploits both variations to identify demand. According to the authors results, a good deal of such variation is within clusters. I believe the authors can do a better job at explaining this and how this is incorporated in the results.

Response: We thank Dr. Paraje for this comment. Indeed, the main source of variation for Deaton's method is the between-village variation in unit values that is largely exogenous – Deaton refers to this as the main identifying assumption. On the other hand, Dr. Paraje is right that some portion of the variation in unit values is a result of endogenous decisions that households are making about quality – what he refers to as quality shading. And a final source of variation is due to measurement error as households incorrectly recall expenditures and/or quantities. Our results incorporate all the three sources of variation in estimating price elasticities.To make the point that Dr. Paraje is referring to more explicit, we have now revised the manuscript and added the following sentence in the Method section on page 8: "It is also important to note that some portion of the variation in unit values comes from these choices in quality that households are making. This variation is also exploited below in deriving our elasticities."

2. In page 6, the authors say that with quality shading price elasticity of demand will be overestimated. Is that so? Let's assume for instance that a change in price of a cigarette expensive brand, widely smoked, leads to the down-trading to a cheaper brand but the quantity of cigarettes smoked remains the same. Basically, that would mean that the estimated elasticity is close to zero, though it has been a change in behaviour. Is elasticity overestimated or underestimated in such a case? May be the authors can answer this question and use this example to make this point clearer.

Response: We thank Dr. Paraje for this comment. To explain why the elasticity is overestimated with quality shading, we make reference to the standard formula for calculating elasticities. Recall that the formula for the price elasticity of demand, , is given as:

where and are the changes in quantity and price respectively. With quality shading, the shifting to a cheaper brand means that the numerator in the formula () hardly changes given that is small. On the other hand, quality shading also implies that the change in price is smaller than it would otherwise be if there was no quality shading. In other words, the consumer, by choosing the cheaper brand, has avoided a huge change in price and therefore is smaller than it would otherwise be. Given that the numerator in the elasticity formula is smaller than it would otherwise be, the calculated elasticity is therefore bigger (in absolute size) than it would otherwise be. In other words, the price elascticity is overestimated.

Here is Deaton himself describing why quality shading results in an overestimated elasticity: "[Any presence of quality shading] will cause price responses to be (absolutely) overstated. The argument is straight forward and depends on higher prices causing consumers to shade down quality. Suppose that when there is a price increase, consumers adapt, not only by buying less, but also by buying lower-quality items, thus spreading the consequences of the price increase over more than one dimension. We want to measure the price elasticity, which in an idealized environment would be calculated as the percentage reduction in quantity divided by the percentage increase in price [the equation above]. However, we observe not the price but the unit value. With quality shading, the unit value increases along with the price, but not by the full amount. As a result, if we divide the percentage decrease in quantity by the percentage increase in unit value, we are dividing by something that is too small, and the result of the calculation will be too large." (Deaton, 1997, p291 – 292)

<u>References</u>

Deaton A. *Analysis of Household Surveys*. Baltimore: Johns Hopkins Press 1997

3. Just after that claim, the authors say that unit values are not the same as prices because of measurement errors (by the way, equation (1) is not an equation: it's an identity). I do not think this is correct. Unit values are different from prices even if everything is recorded perfectly. The main difference between them is that households are price-takers but can choose unit values of the goods they choose to buy. Prices are exogenous to households' choices; unit values are endogenous. That's the main difference (Deaton specifically states this) and, unless I am wrong, the authors should change this. They say: "Households are unlikely to correctly recall the amount of money spent on cigarettes and/or the quantity consumed. In some cases, the survey enumerator might incorrectly

capture this information.” The same could be said about price recording. One could ask about quantities and prices and there would be measurement errors.

Response: Dr. Paraje is correct that equation (1) strictly speaking should be referred to as an identity. We have therefore re-written it as follows with the to signify that it is an identity and not an equation:

.

On the second part of his comment, Deaton does specifically say that unit values are not the same thing as prices because of two reasons: quality shading (described above) and measurement error. Here is Deaton writing in 1997: “Quality effects in unit values are not the only reason why they cannot be treated as prices, and may not even be the most important. Because unit values are derived from reported expenditures and quantities,measurement error in quantity [and expenditure] will be transmitted to measurement error in the unit value, inducing a spurious negative correlation” (Deaton, 1997, p292).

Further, here is Deaton writing in 1988: “In surveys where households report both expenditures and physical quantities, it is possible to divide one by the other to obtain unit values. These unit values, which depend on actual market prices, suggest that there is substantial spatial variation in prices in many developing countries, a finding that makes good sense in the presence of high transport costs. However, it is not possible to use unit values as direct substitutes for true market prices in the analysis of demand patterns. Consumers choose the quality of their purchases, and unit values reflect this choice. Moreover, quality choice may itself reflect the influence of prices as consumers respond to price changes by altering both quantity and quality. Measured unit values are also contaminated by errors of measurement in expenditures and in quantities and are likely to be spuriously negatively correlated with measured quantities” (Deaton, 1988, p418 – 419).

References

Deaton A. Quality, Quantity, and Spatial Variation of Price. *American Economic Review* 1988; 78: 418 – 430

Deaton A. *Analysis of Household Surveys*. Baltimore: Johns Hopkins Press 1997

4. On page 8, they say: “Knowing the pattern of the quality effects (i.e. the magnitude of β), allows us to correct our final price elasticity estimates”. Usually, parameters associated with “quality” are associated with the income elasticity of demand. How is β associated with price elasticity? It’s not obvious at all, from the equation of price elasticity (7). Please, clarify this point.

Response: The parameter that allows one to assess whether quality effects are present in the data, at least within the context of Deaton’s method, is the coefficient on the logarithm of expenditure in a regression of the logarithm of unit values on the logarithm of household expenditure. In other words, it is the coefficient on household expenditure in the so-called unit value regression. This is what Deaton refers to as the expenditureelasticity of quality. In our case, this expenditure elasticity of quality is captured by β. And knowing the magnitude of β allows us to avoid the pitfall of having an overestimated price elasticity as per the discussion of quality shading in point 2 above. In other words, it allows us to correct for a possible overestimate of the price elasticity of demand. This is what links β to the estimate of the price elasticity of demand as per equations (7 to 10) in the manuscript. I refer the reviewer to the discussion of this very point in Deaton (1997) from pages 288 to 292 and again from pages 294 to 299.

References

Deaton A. *Analysis of Household Surveys*. Baltimore: Johns Hopkins Press 1997

5. In addition, why there is no estimation of the income elasticity of demand? I believe it would be very important to have this estimate or to justify why is missing.

Response: Dr. Paraje is indeed correct in his observation that the absence of the income elasticity of demand is conspicuous by its absence. We have since added a discussion of the income elasticity of demand (strictly known as the expenditure elasticity of demand in this case because we are using expenditure data and not income). That discussion now appears in the Method section (as equation 3.11) and in the results section as Table6.

6. On page 8, further below, they claim: “Because of the assumption that prices are fixed within clusters and the fact that we do not have price data, prices are proxied by cluster fixed effects”. Again, my understanding of Deaton’s AIDS tells me this interpretation is wrong. Prices are proxied by unit values and household characteristics. Fixed effects capture tastes, preferences and idiosyncratic factors, as they authors claim right after that sentence. If fixed effects capture prices, tastes, preferences, etc; how is it possible to untangle the price from the other variables to estimate elasticity? I might be wrong, but anyway this needs to be explained better.

Response: Dr. Paraje is correct that our description of why cluster fixed effects are included is not correct. We have since deleted the following sentence from the manuscript: “Because of the assumption that prices are fixed within clusters and the fact that we do not have price data, prices are proxied by cluster fixed effects”.

The only sentences that pertain to the cluster fixed effects now read: “The cluster fixed effects allow us to control for cluster-level tastes and preferences. Similar tastes and preferences are to be expected for narrowly constructed clusters such as villages.”

7. Another question is: given that they cannot estimate a “participation elasticity” by using households that choose to buy cigarettes and those who choose not to buy, is there any self-selection bias in the sample? Why not using, for instance, Heckman’s method to correct for that? Please, clarify why this is not done.

Response: There might be an aspect of selection bias in the sample. Sadly, the dataset that we use for our analysis does not contain any information about households that do not purchase cigarettes. In other words, wedo not know anything about non-purchasing households (nothing about their expenditure, household characteristics, etc…). In other words, the non-purchasing households do not exist in our dataset and therefore we cannot use Heckman’s method. The best we can do is estimate conditional elasticities, a point we make in the manuscript and whose limitations we explicitly acknowledge.

8. Table 1 could also inform the number of clusters (or the proportion of them out of the total) that have households used in the final estimation. If there is a large proportion of them that are not used, the authors should discuss the implications of this. In addition, it would be nice to understand why the average proportion of households per cluster is so low. Usually clusters are a set of blocks or small neighbourhoods or even villages. Less than two households per cluster seems a very small amount. Is this correct? Is there any implication of this in the results (see comment just below)?

Response: This is well noted comment from Dr. Paraje. We have now included a new summary statistic in Table 1 that tells how many clusters there were in total in the survey and how many we actualy use for the analysis. The former is simply referred to in the table as “Total number of clusters” and the latter as “number of effective clusters”. For 2005, the total number of clusters was 322 and the number of clusters actually used in the analysis (effective clusters) is 178. For 2009, the number of clusters was 318 and the number used in the analysis 121. Ideally we would want to use all the

clusters in the analysis given that the Deaton estimator relies on the number of clusters for its consistency properties. However, we cannot use clusters that do not have even a single household purchasing cigarettes and therefore these clusters have to be removed from the analysis(Deaton, 1997). And given that cigarettes are not a commonly consumed commodity in Uganda, there is going to be a preponderance of clusters without a single household purchasing cigarettes. The major limitation coming out of this is that our elasticity estimates might be influenced by clusters having extreme values (i.e outliers). We address this possibility in the Robustness section of the paper where we remove from our analysis any unit values that are extreme using the approach proposed by Guindon et al (2011). The results are not driven by outliers and therefore not sensitive to the small nature of the sample.

Coming to the second issue of very low cluster sizes (i.e number of households per cluster), it is not surprising as households that consume cigarettes are in anyway going to be very few in Low and Middle Incomecountries and Uganda is no exception. However, the issue of small cluster sizes does not appear to be fatal when using Deaton's method. Deaton (1997) argues that the consistency properties of the price elasticity estimator depend on the number of clusters and not on the number of households per cluster. In other words, his estimates converge on the true population parameter when the number of clusters increases and not when the number of households per cluster increases. This is a consequence of the fact that his method relies on between-cluster variation and not on within-cluster variation. So the number of clusters is much more important than the number of households per cluster (cluster size). Deaton (1990; 1997) shows, via a series of Monte Carlo experiments, that his proposed estimator performs very well even with very few observations per cluster. In his own estimates of price elasticities for various commodities in Cote D'Ivoire (Deaton, 1988, 1997), his average cluster size is 1.9 implying that there are some clusters with only one household. He does not flag this asn issue given the aforementioned results of the Monte Carlo exercises discussed above.

References

Deaton A. Quality, Quantity, and Spatial Variation of Price. *American Economic Review* 1988; 78: 418 – 430

Deaton A. Price elasticities from Survey Data: Extensions and Indonesian Results. *Journal of Econometrics* 1990; 44: 281 – 304

Deaton A. *Analysis of Household Surveys*. Baltimore: Johns Hopkins Press 1997

Guindon EG, Nandi A, et al. Socioeconomic differences in the impact of smoking tobacco and alcohol prices on smoking in India. National Bureau of Economics Research 2011; Working Paper Number 17580

9. Table 5 shows the results with bootstrapped standard errors. How many times was the sample bootstrapped? Was the bootstrap made for the whole sample or was made considering some clustering of households? Any implication of the latter?

Response: The sample was bootstrapped 1000 times. The bootstrap was done for the entire sample but at the level of clusters because identification in Deaton's method is sourced at the cluster level. This is Deaton writing about the bootstrapping technique: "The bootstrapped "standard errors" are calculated by making 1,000 draws from the cluster (second-stage) data, recalculating the estimates for each, and then finding (half) the length of the interval that is symmetric around the bootstrapped mean, and that contains 68.3 percent of the bootstrapped estimates" (Deaton, 1997, p317).

References

Deaton A. *Analysis of Household Surveys*. Baltimore: Johns Hopkins Press 1997

Reviewer: 2
Reviewer Name: Nigar Nargis
Institution and Country: American Cancer Society, United States
Please state any competing interests or state ‘None declared’: None declared.

Please leave your comments for the authors below
This is a well written paper and makes important contribution to the economics of cigarette smoking in the context of Sub-Saharan Africa. It uses a household panel survey to estimate price elasticity of conditional cigarette demand in Uganda.

While the study makes the best use of available data in applying Deaton's model, authors need to make further clarifications to the methods of data analysis and recognize the limitations of the study more clearly.

1. Table 1 shows average household expenditure decreases in real terms. Is it consistent with overall economic growth in the country?

Response: The Ugandan economy did grow between 2005 and 2009. For example, per capita GDP growth averaged 5% between these two periods. On the other hand, inflation was relatively high. Between 2005 and 2009, the average inflation rate was 9%. This means the average rate of inflation was greater than the average rate of growth in incomes. This explains the decline in real household incomes/expenditure between the two periods.

2. There are less than two households per cluster on average. What is the range of the number of households by cluster? I am guessing that some clusters may have a single household. It can cause a limitation in using spatial variation in price for the identification of the effect of price changes. Because in these cases the cluster unit value is essentially the unit value of a household, which can hardly represent a full cluster. In fact, the finding in ANOVA that about 30% residual variation in unit value is due to within cluster variation might be an underestimate because of the representation of some clusters with single households. For these clusters, 100% variation comes from between cluster variation and cluster fixed effects cannot be estimated in equation 3.

Response: This is a very well noted issue from Dr. Nargis. The range of households per cluster runs from 1 to a maximum of 6. This is not surprising as households that consume cigarettes are in anyway going to be veryfew in Low and Middle Income countries and Uganda is no exception. However, the issue of small cluster sizes does not appear to be fatal when using Deaton’s method. Deaton (1997) argues that the consistency properties of the price elasticity estimator depend on the number of clusters and not on the number of households per cluster. In other words, his estimates converge on the true population parameter when the number of clusters increases and not when the number of households per cluster increases. This is a consequence of the fact that his method relies on between-cluster variation and not on within-cluster variation. So the number ofclusters is much more important than the number of households per cluster (cluster size). Deaton (1990; 1997) shows, via a series of Monte Carlo experiments, that his proposed estimator performs very well even with very few observations per cluster. In his own estimates of price elasticities for various commodities in Cote D’Ivoire (Deaton, 1988, 1997), his average cluster size is 1.9 implying that there are some clusters with only one household. He does not flag this as an issue given the aforementioned results of the Monte Carlo exercises discussed above.

References

Deaton A. Quality, Quantity, and Spatial Variation of Price. *American Economic Review* 1988; 78: 418 – 430

Deaton A. Price elasticities from Survey Data: Extensions and Indonesian Results. *Journal of Econometrics* 1990; 44: 281 – 304

Deaton A. *Analysis of Household Surveys*. Baltimore: Johns Hopkins Press 1997

3. What is the retention rate of the panel of households in 2009? The numbers of households and clusters reduced significantly between 2005 and 2009. Can it create any systematic bias in the analysis? Does it have to do with the lack of spatial variation in prices observed in the 2009 sample in the ANOVA exercise? What other factor variables were used in ANOVA?

Response: Given Dr. Nargis' very important points made in point 6 and point 7 below about the complications introduced with using Deaton's method within the context of a panel, we have decided to remove the parts of the paper that relate to panel analyses/panel estimations. In this way, there is no longer a need to worry about the retention rate given we treat the years (2005 and 2009) as separate cross-sections.

4. How large are the samples of pack purchases and stick purchases? Authors can test if pack versus stick purchases can contribute to the variation in prices by using it as a factor variable in ANOVA.

Response: Stick purchases often tend to predominate in many Low and Middle Countries and Uganda is not an exception. However, Deaton's method requires standardized units for the analysis. This is seen in the fact that the main variable, the unit value, is defined as the amount paid per standardized unit. Therefore, the analysis has to be performed either in terms of sticks or in terms of packs but not both. The results are equivalent whichever one chooses. Further, the ANOVA is a one-way ANOVA with only one outcome variable (unit values) and one independent categorical variable (clusters). In other words, the one-way ANOVA is equivalent to running a regression of unit values on cluster dummies (Deaton, 1997, p286). Given the one-way nature of the ANOVA exercise, one cannot include more factor variables beyond the cluster variable.

Reference

Deaton A. *Analysis of Household Surveys*. Baltimore: Johns Hopkins Press 1997

5. In Table 3, the coefficient of Year indicates that unit values were lower in 2005 than in 2009 according to the description of the Year dummy variable in the Notes: Year takes on the value of 1 if the household was surveyed in 2005 and zero if surveyed in 2009. In contrast, the average price level shown in Table 1 was higher in 2005 than in 2009. Please double check if the base year used in the regression was 2005.

Response: We thank Dr. Nargis for spotting this important typographical error in the notes to Table 3. The correct note relating to the year dummy should read: "Year takes on the value of 0 if the household was surveyed in 2005 and 1 if surveyed in 2009". Therefore, the base year is 2005. However, given Dr. Nargis' important points in point 6 and point 7 below about the complications of estimating Deaton's method within the context of panel datasets, there's no longer a need to include year dummies in our analysis as we now conduct the analysis separately for 2005 and 2009. In other words, we no longer conduct the analysis nor report results for the pooled sample. Therefore, the new tables in the paper do not contain any notes relating to the year dummy.

6. Did you try to estimate the model using panel data regression on pooled dataset that would control for household level fixed effects? It would be worth comparing the results with the pooled regression estimates. Please explain the merit of using pooled OLS instead of fixed effects model.

Response: This is a very important point from Dr. Nargis. Dr. Nargis is correct that it does not make much econometric sense to estimate Deaton's method within a panel set-up. Deaton himself does not discuss nor point to the possibility of using his method to estimate elasticities within a panel set-up. This is something that we overlooked in our analysis and Dr. Nargis' comment forced us to read

Deaton more closely. We have, therefore,decided to restrict our analysis to only cross sections. In other words, we have decided to estimate elasticities separately for 2005 and 2009 treating them as separate cross sections and done away with conducting the analysis on the pooled sample. This then means we now have to rewrite the section on Robustness using either the 2005 or 2009 sample as opposed to previously where it only focused on the pooled sample.

7. In the pooled regression, the error terms of the same household are expected to be correlated. Did you correct standard errors of estimates for the correlation of repeated observations?

Response: This is another important point raised by Dr. Nargis. Our response here is identical to the one given in point 6 above. In the initial analysis we did not correct for the possible correlation in error terms – something that we overlooked. Given that it is impossible to do this within the context of Deaton's method (Deaton developed his technique to only deal with cross sectional data), we have decided to remove from the paper any discussion or analysis of the pooled sample given the point raised by Dr. Nargis.

Reviewer: 3
Reviewer Name: Christian Schafferer
Institution and Country: Department of International Trade Overseas Chinese University Taiwan

Please state any competing interests or state 'None declared': None declared

Please leave your comments for the authors below
Review

This paper provides estimates of price elasticity of demand for cigarette consumption in Uganda. Publication certainly is in the interest of the scientific community: 1) Studies on price elasticity estimates for Uganda are rare. 2) The authors apply an alternative estimation method that may deliver more accurate results for African countries. The paper thus certainly deserves attention. However, several points should be addressed before publication.

Abstract/Strengths and Limitations

1. P3/7: I am not sure whether it is important to mention that Angus Deaton received the Nobel Prize.

Response: We thank Dr. Schafferer for this observation. The part referring to Deaton's Nobel Prize has been edited out of the Strengths and Limitations section.

2. P3/14-17: Considering only cigarette demand is quite common, and in most cases do not constitute a major limitation unless cigarette use is rare in comparison to other tobacco products. If so, this should be mentioned in the introduction/discussion section of the paper.

Response: Duly noted comment from Dr. Schafferer. We have edited out of the Strengths and Limitations section the part referring to the consideration of cigarette demand as a limitation.

3. Introduction
The introduction could cover more details regarding the smoking prevalence in African countries, price elasticity figures and about the study itself. That is, a literature review is missing. Why is smoking prevalence higher than in other African countries? How about cigarette prices/availability? P4/28-47 Elasticity estimates: Which countries/geographic areas are covered here? Any difference between developed and less-developed states? Why do estimates differ? Because of different methodologies?
P4/52-P5/10: Although studies are rare for Africa (Uganda), there is some (comparative) literature (e.g. Li-Ming Ho et. al, Int J Public Health (2017) 62: 899). What estimates have been previously

reported for African countries? Do we expect Uganda do be different? What is so special about Uganda (or perhaps for the whole of Africa) so that the application of Deaton's methodology is necessary/more promising. I am aware that the authors state several reasons in the Method section of the paper, but it would be beneficial to the reader to get an overall idea about what the paper has to offer in the Introduction section. So far, this is not mentioned clearly enough in the Introduction. Perhaps some of the explanations in the Method section can be moved to/highlighted in the Introduction.
Perhaps the authors could also mention whether their methodology has previously been applied in other studies on cigarette price elasticity. If so, what are the results? Any positive experience?

Response: Dr. Schafferer raises good points here. We have since rewritten much of the introduction section to satisfy the comments raised here. We have included a mini-literature review in addition to including adiscussion of the paper by Li-Ming Ho, Christian Schafferer, et al. (2017). We were unaware of this paper at the time of writing and we thank Dr. Schafferer for pointing it to us.

4. Method
This section of the paper is quite extensive. Perhaps this section should be used to focus on the technical aspects of the methodology, whereas shortcomings etc. could be mentioned/discussed in the Introduction/Discussion section of the paper.
For example, p.26/19-27 dealing with recently emerged literature and its results could be moved to the Discussion section.
Moreover, some efforts could be made to improve the 'flow' of the paper.

Response: We acknowledge the comment raised here by Dr. Schafferer. However, our description of Deaton's method here is far less technical and far less extensive than contained in Deaton's own books and papers and other studies that have implemented the method for cigarettes. Moreover, given that one of our paper's main contributions is to "introduce" the method to an African audience of researchers, we think it proper to give the Method section a complete treatment as has been done in the paper. In addition, 2 of the reviewers of the present paper have here and there recommended that we include some additional details to this section to add even more clarity.

However, we have moved the description of other studies using Deaton's method , as per Dr. Schafferer's advice, to the Introduction and the Discussion section.

5. Discussion
This section needs greater attention. It looks more like a summary of key features/results of the study. It lacks a discussion about the problem the study endeavours to solve. Perhaps it should discuss the problem of increased smoking prevalence in Uganda and what role taxation could play in reducing cigarette use by referring to the experience of other African countries. Furthermore, it should probably discuss the question of why the applied methodology was chosen and whether expectations were met. Do the authors recommend the future application of the Deaton method? How might such results differ from other methodologies? Again, this may be done by including previously published studies. How effective may taxation be in Uganda considering socio-political and institutional circumstances? Moreover, apart from discussing the limitations of the study, it may be helpful to the reader to understand how future research on cigarette price elasticity could be improved.

Response: We thank Dr. Schafferer for this suggestion. We have since re-written the Discussion section along the lines suggested by him. The Discussion section now reads better than before.

VERSION 2 – REVIEW

**REVIEWER**	Guillermo Paraje<br>Universidad Adolfo Ibáñez Chile<br>I am the Principal Investigator for Chile of a project where Dr Corne Van Wallbeck is also a Principal Investigator (for South Africa)
**REVIEW RETURNED**	11-Dec-2018

**GENERAL COMMENTS**	The authors have been very clear and solid in addressing my comments. I have no further comments.

**REVIEWER**	Nigar Nargis<br>American Cancer Society, United States
**REVIEW RETURNED**	13-Dec-2018

**GENERAL COMMENTS**	The authors have done a good job in addressing my comments on the panel data aspects of the analysis by dropping the pooled regression analysis and basing the conclusions on cross-sectional analysis of each year's data.<br><br>I have one additional comment. They have added the expenditure elasticities in the revised version. It is not appropriate to label cigarettes as normal good based on these estimates which are statistically insignificant.

**REVIEWER**	Christian Schafferer<br>Department of International Tradeb Overseas Chinese University Taiwan
**REVIEW RETURNED**	26-Dec-2018

**GENERAL COMMENTS**	The authors have made substantial improvements. The revised manuscript is well-written and clear. It offers valuable data and suggestions regarding the application of Deaton’s method. I thus recommend its publication.

VERSION 2 – AUTHOR RESPONSE

Reviewer(s)' Comments to Author:

Reviewer: 1
Reviewer Name: Guillermo Paraje
Institution and Country: Universidad Adolfo Ibáñez, Chile
Please state any competing interests or state 'None declared': I am the principal investigator in a project with the participation of Prof Van Wallbeeck (also PI)

Please leave your comments for the authors below
The authors have been very clear and solid in addressing my comments. I have no further comments.

Reviewer: 2
Reviewer Name: Nigar Nargis
Institution and Country: American Cancer Society, USA
Please state any competing interests or state 'None declared': None declared.

Please leave your comments for the authors below
The authors have done a good job in addressing my comments on the panel data aspects of the analysis by dropping the pooled regression analysis and basing the conclusions on cross-sectional analysis of each year's data.

I have one additional comment. They have added the expenditure elasticities in the revised version. It is not appropriate to label cigarettes as normal good based on these estimates which are statistically insignificant.

RESPONSE: Dr. Nargis is correct in this observation. We have now rewritten the sentence pertaining to the expenditure elasticity this way:

The estimates in Table 6 are imprecisely estimated and are, therefore, statistically insignificant.

Reviewer: 3
Reviewer Name: Christian Schafferer
Institution and Country: Department of International Trade - Overseas Chinese University - Taiwan
Please state any competing interests or state 'None declared': None declared

Please leave your comments for the authors below
The authors have made substantial improvements. The revised manuscript is well-written and clear. It offers valuable data and suggestions regarding the application of Deaton's method. I thus recommend its publication.
